# Revisiting the expression of BDNF and its receptors in mammalian development

**DOI:** 10.3389/fnmol.2023.1182499

**Published:** 2023-06-22

**Authors:** Eli-Eelika Esvald, Jürgen Tuvikene, Carl Sander Kiir, Annela Avarlaid, Laura Tamberg, Alex Sirp, Anastassia Shubina, Florencia Cabrera-Cabrera, Arno Pihlak, Indrek Koppel, Kaia Palm, Tõnis Timmusk

**Affiliations:** ^1^Department of Chemistry and Biotechnology, Tallinn University of Technology, Tallinn, Estonia; ^2^Protobios LLC, Tallinn, Estonia; ^3^dxlabs LLC, Tallinn, Estonia

**Keywords:** BDNF, *TrkB*, p75NTR, development, evolution, RNA-Seq, Western blot

## Abstract

Brain-derived neurotrophic factor (BDNF) promotes the survival and functioning of neurons in the central nervous system and contributes to proper functioning of many non-neural tissues. Although the regulation and role of BDNF have been extensively studied, a rigorous analysis of the expression dynamics of *BDNF* and its receptors *TrkB* and *p75NTR* is lacking. Here, we have analyzed more than 3,600 samples from 18 published RNA sequencing datasets, and used over 17,000 samples from GTEx, and ~ 180 samples from BrainSpan database, to describe the expression of *BDNF* in the developing mammalian neural and non-neural tissues. We show evolutionarily conserved dynamics and expression patterns of *BDNF* mRNA and non-conserved alternative 5′ exon usage. Finally, we also show increasing BDNF protein levels during murine brain development and BDNF protein expression in several non-neural tissues. In parallel, we describe the spatiotemporal expression pattern of BDNF receptors *TrkB* and *p75NTR* in both murines and humans. Collectively, our in-depth analysis of the expression of *BDNF* and its receptors gives insight into the regulation and signaling of BDNF in the whole organism throughout life.

## Introduction

BDNF is a member of the neurotrophic family of secreted proteins and has well-established roles in the nervous system, e.g., in neuronal survival and differentiation, synapse formation and maturation, and development of neural circuits (Park and Poo, 2013; Wang et al., 2022). Moreover, BDNF is associated with numerous neuropsychiatric disorders, such as depression, bipolar disorder, schizophrenia, addiction, and various neurodevelopmental conditions (Autry and Monteggia, 2012; Wang et al., 2022). In recent years the functions of BDNF have been demonstrated in various non-neural tissues, including the heart (Donovan et al., 2000; Fulgenzi et al., 2015; Li et al., 2022), lung (Paris et al., 2020), skin (Rutlin et al., 2014), mammary gland (Liu et al., 2012; Sar Shalom et al., 2019), and skeletal muscle (Delezie et al., 2019; Ahuja et al., 2021). Furthermore, BDNF regulates blood insulin levels (Yang et al., 2019; Fulgenzi et al., 2020), and BDNF signaling is important in kidney development (García-Suárez et al., 2006; Endlich et al., 2018) and serves as a potential marker for chronic kidney disease (Afsar and Afsar, 2022). In humans, BDNF also contributes to platelet aggregation (Boukhatem et al., 2021). Taken together, BDNF signaling is important in both the nervous system and in non-neural tissues, highlighting the importance of understanding its developmental regulation.

BDNF protein is synthesized as a prepro-precursor protein into the endoplasmic reticulum, with the pre-region cleaved co-translationally, resulting in proBDNF. proBDNF is processed to mature BDNF protein either in the Golgi apparatus (Mowla et al., 1999, 2001) or extracellularly (Lee et al., 2001). Both proBDNF and mature BDNF form non-covalently associated dimers and bind two types of receptors, TrkB and p75NTR, and therefore affect cells in a diverse way (Lu et al., 2005). The TrkB receptor is a member of the tropomyosin-related kinase family of tyrosine kinases and binds either mature BDNF or NT-4 (Reichardt, 2006). Binding of neurotrophins causes TrkB dimerization and activation of Ras-MAPK, PI3K-Akt, and PLCγ1-IP3/DAG signaling pathways (Reichardt, 2006). The TrkB protein-encoding gene *NTRK2* encodes several TrkB isoforms generated by alternative splicing, and the main isoforms in humans are the full-length TrkB isoform (hereafter TrkB-FL), and the C-terminally truncated isoforms TrkB-T1 and TrkB-Shc, both of which lack the tyrosine kinase domain, though the latter retains the Shc-binding site (Stoilov et al., 2002; Luberg et al., 2010). While the TrkB-T1 isoform sequesters both BDNF and TrkB-FL (Haapasalo et al., 2002) and therefore functions as a dominant-negative TrkB isoform, it can also elicit intracellular signals on its own, e.g., to regulate calcium influx in glial cells and cardiomyocytes (Tessarollo and Yanpallewar, 2022). Similarly, the TrkB-Shc isoform acts as a dominant-negative regulator of TrkB signaling as the TrkB-FL does not phosphorylate TrkB-Shc after dimerization (Stoilov et al., 2002). The p75NTR receptor is a member of tumor necrosis factor receptor superfamily and binds all neurotrophins – NGF (Sutter et al., 1979), BDNF (Rodriguez-Tebar et al., 1990), NT-3 (Rodríguez-Tébar et al., 1992), and NT-4 (Rydén et al., 1995). The p75NTR receptor preferentially binds proneurotrophins, whereas TrkB preferentially binds mature neurotrophins (Reichardt, 2006). Following proneurotrophin binding, p75NTR activates NF-kB, RhoA, and Jun kinase pathways which promote neuronal survival, inhibit neurite growth, or induce apoptosis, respectively (Reichardt, 2006). During development p75NTR signaling induces normal cell death, while in adults it triggers apoptosis after injury (Kraemer et al., 2014). In summary, the different effects of BDNF on target cells and tissues depend on the expression of specific TrkB isoforms and the p75NTR receptor.

The genes encoding neurotrophins are highly conserved throughout vertebrates, and they have been proposed to originate from a single ancestral gene (Hallböök et al., 1991; Hallböök, 1999). In murines the *Bdnf* gene contains eight 5′ non-coding exons that are controlled by distinct promoter regions and are spliced to the common protein-coding 3′ exon (Timmusk et al., 1993b; Aid et al., 2007). The activity-dependent subcellular localization of *Bdnf* transcripts in neurons has been thoroughly studied (Tongiorgi et al., 1997; Pattabiraman et al., 2005; Chiaruttini et al., 2008, 2009; Baj et al., 2013, 2016; Singer et al., 2018; Colliva and Tongiorgi, 2021). Moreover, studies in mice have shown that different *Bdnf* transcripts regulate dendrite complexity (Maynard et al., 2017) and impairing the expression of specific *Bdnf* transcripts has distinct functional consequences. For example, loss of *Bdnf* exon I-containing transcripts results in increased body weight, reduced thermogenesis (You et al., 2020), heightened aggression in male mice (Maynard et al., 2016), and impaired maternal care (Maynard et al., 2018). In contrast, loss of *Bdnf* exon IV transcripts affects the development of inhibitory synapses (Hong et al., 2008; Sakata et al., 2009), disrupts sleep and sensory information processing, and impairs fear memory retrieval (Hill et al., 2016).

Here, we aimed to evaluate the potential role of BDNF throughout mammalian development by revisiting the expression of *BDNF* using bioinformatical analysis of published RNA sequencing datasets. First, we analyzed the spatiotemporal expression of *BDNF* mRNA, including transcripts with different 5′ exons and 3′ untranslated regions (UTRs), in neural and non-neural tissues of different mammals. We also examined the expression of BDNF receptors *TrkB* and *p75NTR*. We then focused on the cell type-specific *BDNF* expression in adult mice and humans using available single-cell sequencing data. Finally, we complemented the bioinformatics data on BDNF expression at the protein level in the nervous system and non-neural tissues during development of three widely used murine animal models: BALB/c and C57BL/6 J mice and Wistar rats. Altogether, the comprehensive description of the expression patterns of *BDNF* and its receptors across different tissues and developmental stages provides a highly valuable resource to the neurotrophin research community.

## Materials and methods

### Bioinformatical analysis

Raw data in fastq format from previously published RNA sequencing datasets (Keane et al., 2011; ENCODE Project Consortium, 2012; Merkin et al., 2012; Fagerberg et al., 2014; Yu et al., 2014; Vied et al., 2016; Li et al., 2017; Söllner et al., 2017; Tabula Muris Consortium, Overall coordination, Logistical coordination, Organ collection and processing, Library preparation and sequencing, Computational data analysis, 2018; Cardoso-Moreira et al., 2019; Luo et al., 2020; Shafik et al., 2021; Rayan et al., 2022), were obtained from EMBL-EBI European Nucleotide Archive database using www.sra-explorer.info. Accession numbers for all datasets are shown in Supplementary Table 1.

Adapter and quality trimming were performed using BBDuk (part of BBMap version 38.90) with the following parameters: ktrim = r k = 23 mink = 11 hdist = 1 tbo qtrim = lr trimq = 10 maq = 10 minlen = 25. Mouse sequencing reads were mapped to mm10 (primary assembly and annotation obtained from GENCODE, release M25, GRCm38), rat sequencing reads were mapped to rn6 (primary assembly and annotation obtained from Ensembl, release 104, RGSC 6.0/Rnor_6.0), human sequencing reads were mapped to hg19 (primary assembly and annotation obtained from GENCODE, release 37, GRCh37), rhesus macaque sequencing reads were mapped to Mmul_10 (primary assembly and annotation obtained from Ensembl, release 108, Mmul_10), rabbit sequencing reads were mapped to OryCun2.0 (primary assembly and annotation obtained from Ensembl, release 108, OryCun2.0) and opossum sequencing reads were mapped to ASM229v1 genome (primary assembly and annotation obtained from Ensembl, release 108, ASM229v1) using STAR aligner (version 2.7.4a) with default parameters. To increase sensitivity for unannotated splice junctions, splice junctions obtained from the 1st pass were combined per dataset and filtered as follows: junctions on mitochondrial DNA and non-canonical intron motifs were removed; only junctions detected in at least 10% of samples (rounded up to the nearest integer) in the whole dataset were kept. Filtered junctions were added to the 2nd pass mapping using STAR aligner. RNA sequencing reads were assigned to features using FeatureCounts (version 2.0.1). The following parameters were used for paired-end data: -p –B –C –J; and single-end data: -J. To count 3’ UTR/exon sequencing reads for *BDNF* and *NTRK2*, a custom SAF file was used (Supplementary Table 2 Custom SAF files). To characterize the total *BDNF,* 5′ exon and 3’ UTR levels throughout development, *NTRK2* 3′ exons and *NGFR* levels, counts per million (CPM) of the assigned RNA sequencing reads were calculated, and normalized with the length of the feature in kb where indicated. To analyze changes in *BDNF* 3′ long UTR proportions during development, CPM of the long 3’ UTR region was length-normalized and divided with the length-normalized CPM of *BDNF* coding sequence (CDS), therefore showing the levels of transcripts with long 3’ UTR from the total *BDNF*. The UCSC Liftover tool was used to convert the coordinates of BDNF coding sequence, 5′ exons and 3’ UTRs from data of human to rhesus macaque, and from mouse to rabbit and opossum genomes.

Meta-analysis of both mouse and rat data was performed to study *Bdnf, Ntrk2* and *Ngfr* expression during development. For analyzing the expression of unique *Bdnf* 5′ exons, length-normalized 5′ exon CPM values were further normalized with length-normalized *Bdnf* CDS CPMs for the corresponding samples. To visualize the distribution of transcripts with *Bdnf* 5′ exons, sums of the normalized 5′ exon values were calculated in each group and divided by the total sum of all normalized 5′ exon values in the group, showing the composition in percentages. The results were visualized using ggplot2 (version 3.3.5) in R (version 4.1.2).

Data mining and visualization was also performed on human Genotype-Tissue Expression project (GTEx) portal gene and exon datasets (dbGaP Accession phs000424.v8.p2), human developmental transcriptome data from BrainSpan (RNA-Seq Gencode v10 summarized to genes and RNA-Seq Gencode v10 summarized to exons), and The Human Protein Atlas^1^ mouse transcript (24. RNA isoform data) and brain subregion gene data (16. RNA mouse brain subregion sample gene data). The human GTEx data used for the analyses were obtained from the GTEx Portal^2^ on 12/01/2021, the human BrainSpan data were obtained from the BrainSpan Atlas of the Developing Human Brain^3^ on 12/01/2021 and the mouse data from The Human Protein Atlas (see footnote 1) were obtained on 13/09/2021. For the pre-analyzed datasets, the level of each 5′ exon was normalized using the sum of all annotated 5′ exons or transcripts containing the respective 5′ exons in the respective sample to calculate the 5′ exon ratios. Then the distribution of 5′ exons usage in *BDNF* transcripts was visualized as described previously.

### Mimotope variation analysis

To determine the epitope of 3C11 anti-BDNF monoclonal antibody (Icosagen, catalogue #327–100), mimotope-variation analysis was used as described previously (Sadam et al., 2018, 2021). Raw peptide counts in each sample were normalized with total peptide count. The 12-mer peptides obtained from the MVA workflow or from sequencing of the input *E. coli* M13 phage library were then aligned to different mouse, rat and human neurotrophin protein sequences (obtained from Uniprot) matching in at least 6 amino acid positions. Alignment loads were calculated as sum of normalized peptide counts aligning to each of the amino acid positions, with each amino acid position counted only once per unique peptide. To reduce the effect of spurious alignment of high-count peptides, the peptide with the highest normalized count was removed from the analysis for each amino acid position. Relative antibody binding at each amino acid position was calculated as fold over the input library alignment load.

### Protein lysates

Animal work was performed as published in Sirp et al. (2022). All experiments concerning animals were performed in agreement with the local ethics committee and European Directive 2010/63/EU. Briefly, BALB/c mouse strain (Envigo), C57BL/6 J (Envigo), and Wistar rats (RccHan:WI, Envigo) were housed in conventional polycarbonate or H-TEMP polysulfone cages (2–4 animals per cage) with *ad libitum* access to clean water and food pellets (ssniff Spezialdiäten, GmbH) under a 12-h light/dark cycle in humidity and temperature-controlled room (temperature 22 ± 1°C and humidity 50 ± 10%).

The female mouse estrous cycle was monitored by visual observations (Champlin et al., 1973) followed by breeding in the evening. The presence of vaginal post-coitum protein plug was confirmed the next morning (no later than 12 h from breeding) and determined as embryonic 0.5 gestational stage. To obtain samples from embryonic stages, the pregnant mothers were euthanized by carbon dioxide inhalation, pups were collected, and embryonic brains were dissected in ice-cold 1× phosphate-buffered saline (PBS) solution. Postnatal (P) 0 stage was determined as the day of the birth and all other ages followed accordingly. Samples were collected from both female and male animals. To collect postnatal brain samples, rats and mice were killed by cervical dislocation and decapitated with a guillotine.

Protein lysates were prepared as described in Sirp et al. (2022). Briefly, tissue samples were dissected in ice-cold 1× phosphate-buffered saline (PBS) and stored at −80°C until further use. For each investigated time point, tissue samples from 2 to 3 different animals were combined and homogenized in ice-cold RIPA buffer [50 mM Tris–HCl (pH 8.0), 150 mM NaCl, 1% NP-40, 0.5% Na-deoxycholate, 0.5% sodium dodecyl sulfate (SDS), 1× cOmplete™ Protease Inhibitor Cocktail (Roche)]. For brain and non-neural tissues 7 and 10 μL RIPA per 1 mg of tissue, respectively, was used. All samples were homogenized using tissue grinder PELLET PESTLE® Cordless Motor (Kimble-Chase, DWK Life Sciences), sonicated 15 s with Torbeo Ultrasonic probe sonicator (36810-series, Cole Parmer), and centrifuged at 4°C at 16,000 g for 20 min. Soluble fraction was kept as protein lysate and protein concentration was measured using Pierce™ BCA Protein Assay Kit (Thermo Scientific).

### Western blot

Fifty micrograms of total protein and different amounts of recombinant mature BDNF protein (Icosagen, cat. no P-105-100) were separated on 15% SDS-PAGE gel and transferred to a PVDF membrane using Trans-Blot Turbo Transfer system (Bio-Rad, Mixed MW program [1,3A, 25 V (const), 7 min)]. The membrane was blocked for 1 h at room temperature in 5% skimmed milk in TBST buffer (1× Tris-buffered saline (pH 7.4) and 0.1% Tween-20), incubated overnight at 4°C with primary anti-BDNF antibody (Icosagen, cat. no 327–100, clone 3C11, 1 mg/mL, 1:1000) in 2% milk-TBST, and overnight at 4°C with secondary antibody anti-mouse IgG conjugated with horseradish peroxidase (Thermo Fisher Scientific, 1:5000) in 2% milk-TBST. After both incubations, the membrane was washed 3 times with TBST for 5 min at room temperature.

Chemiluminescence signal was produced with SuperSignal™ West Femto or Atto Maximum Sensitivity Substrate (Thermo Fisher Scientific) and measured using imaging system ImageQuant Las 4,000 (GE Healthcare Life Sciences). For loading control, the membrane was stained with Coomassie solution (0.1% Coomassie Brilliant Blue R-250 Dye, 25% ethanol, 7% acetic acid), followed by washes with destaining solution (30% ethanol, 10% acetic acid) and rinsing with tap water. The membrane was imaged using ImageQuant Las 4,000 (GE Healthcare Life Sciences).

BDNF protein levels were quantified using densitometric analysis on ImageQuant TL software (GE Healthcare Life Sciences). As indicated in the figure legend, the amounts of BDNF protein in the tissue lysates were calculated based on the calibration curve of recombinant BDNF protein.

### CRISPR interference-mediated silencing of *Bdnf* expression

The preparation and growing of Sprague Dawley rat primary cortical neurons was performed as described in Esvald et al. (2022). The used guide RNA (gRNA) sequences targeting either *Bdnf* promoters I or IV (pI or pIV), or control gRNA were as follows: *Bdnf* pI gRNA 1 5’-GTCACGTAACTGGCTCAGAG-3′, *Bdnf* pI gRNA 2 5’-GCCCTAGCCTGACAAGGCGA-3′, *Bdnf* pIV gRNA 1 5’-GCACTAGAGTGTCTATTTCG-3′, *Bdnf* pIV gRNA 2 5’-GATTTCATGCTAGCTCGCCG-3′, control gRNA 5’-GCTGATCTATCGCGGTCGTC-3′. Lentiviruses encoding the gRNAs or dCas9-KRAB were generated as described in Esvald et al. (2022). Neurons were infected with the indicated lentiviruses on the day of plating, half of culture media was changed at 2 days *in vitro* (DIV) and 5 DIV. A final concentration of 10 μM FDU (Sigma) was added to the media from 2 DIV. At 7 DIV, spontaneous neuronal activity was suppressed by adding 1 μM tetrodotoxin (Tocris Bioscience), and the cells were treated with 25 mM KCl (with 5 μM D-APV (Cayman Chemical Company) to reduce excitotoxicity) for 6 h at 8 DIV. RNA extraction, cDNA synthesis and qPCR, and protein extraction were conducted as described previously in Esvald et al. (2022). Protein concentration was measured using Pierce BCA Protein Assay kit (Thermo Scientific) and 15 μg of total protein was loaded on the gel along with bacterially expressed recombinant proBDNF (Alomone labs, cat. no B-257) and recombinant mature BDNF (Icosagen).

### Overexpression of BDNF-V5 in HEK293 cells

Rat *Bdnf* coding sequence was cloned into pcDNA3.1/V5-His-TOPO expression vector (Life Technologies) where the CMV promoter was replaced with EF1α promoter. HEK293 cells were grown in Minimum Essential Medium (MEM, Corning, cat. no 10-010-CV) supplemented with 10% fetal bovine serum (PAN Biotech), 100 U/mL penicillin, and 100 μg/mL streptomycin (Gibco). The cells were transfected on 12-well plate with PEI (Sigma) with 1.5 μg DNA per well using 1:2 DNA:PEI ratio. Cells were lysed 24 h post transfection directly into 1x Laemmli buffer (containing 5% β-mercaptoethanol) and proteins were subjected to SDS-PAGE and Western blot using anti-V5 antibody (Thermo Fisher Scientific, #R960-25, 1:5000) or anti-BDNF 3C11 antibody.

## Results

### *Bdnf* mRNA levels in murine brain during development

**Figure 1:**
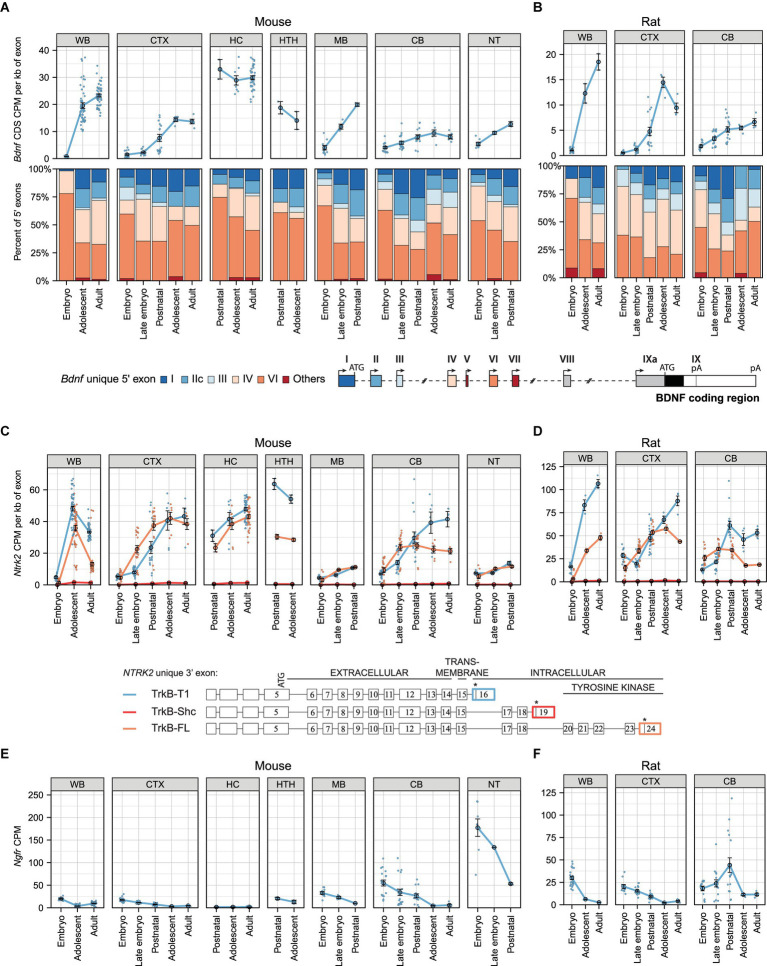
The expression levels of mRNAs encoding BDNF and its receptors TrkB (*Ntrk2* mRNA) and p75NTR (*Ngfr* mRNAs) during murine development in different brain regions. Meta-analysis of *Bdnf*, *Ntrk2* (encoding TrkB) and *Ngfr* (encoding p75NTR) expression levels in mouse **(A,C,E)** and rat **(B,D,F)** brain regions throughout development. Levels in embryo (E10.5-E14.5/E11-E15 mouse/rat), late embryo (E15.5-E18.5/E17-E20 mouse/rat), postnatal (P0-P14), adolescent (P22-56) and adult (P62+) animals are shown. **(A,B)** Total *Bdnf* expression measured by levels of *Bdnf* coding sequence (CDS, upper panel) and distribution of *Bdnf* 5′ exons (lower panel) are shown as depicted on the schematics of murine gene structure with different colors. The exons indicated with gray color were not included in the analysis of 5′ exons. **(C,D)** The mRNA levels of different *TrkB* isoforms based on the levels of unique 3′ exons of the *Ntrk2* gene (rat or mouse counterpart of human *NTRK2* exons 16, 19, 24). The 3′ exons specific for *TrkB* isoforms *TrkB-T1*, *TrkB-Shc*, and *TrkB-FL* were measured as shown with colors on the schematics [adapted from Luberg et al. (2010)]. Asterisks on the schematics mark stop-codons. **(E,F)** Total *Ngfr* mRNA expression levels. Data from individual animals are shown as small dots, circles indicate mean values and error bars represent standard error of the mean (SEM). All used datasets and underlying data are shown in Supplementary Table 3. WB – whole brain, CTX – cerebral cortex, CB – cerebellum, HC – hippocampus, HTH – hypothalamus, MB – midbrain, NT – neural tube, CDS – coding sequence, CPM – counts per million.

As BDNF is widely studied in murines and it plays an important role in the developing central nervous system, we set out to first describe the spatiotemporal expression pattern of *Bdnf* in mouse and rat development using various publicly available RNA sequencing datasets (listed in Supplementary Table 3). Our analysis shows that total *Bdnf* mRNA levels increase remarkably during development in the whole brain of both mouse (Figure 1A) and rat (Figure 1B). Of the analyzed tissues, the highest levels of *Bdnf* mRNA were detected in the adolescent and adult mouse hippocampus (Figure 1A) and in the adolescent rat cerebral cortex (Figure 1B).

We then analyzed the developmental expression of *Bdnf* transcripts which differ in their 5′ exons. We detected all main *Bdnf* transcripts, with most transcripts containing 5′ exons IV and VI, and a lower proportion of transcripts containing exons I, IIc, and III (Figures 1A,B). *Bdnf* exon I and IIc-containing mRNAs contribute more to total *Bdnf* mRNA levels in the mouse cerebral cortex and hypothalamus and less in the hippocampus (Figure 1A), which is in agreement with previously published contribution of different *Bdnf* transcripts to BDNF protein levels in these brain regions (Maynard et al., 2016). Notably, the relative proportion of *Bdnf* exon I and IIc transcripts generally increases and proportion of *Bdnf* exon VI transcripts slightly decreases during mouse and rat development (Figures 1A,B). In contrast to other brain regions, the proportion of *Bdnf* exon I-containing transcripts decreases during postnatal cerebellar development in both mouse and rat (Figures 1A,B). Taken together, our results show developmental upregulation of total *Bdnf* mRNA levels and specific regulation of *Bdnf* transcripts in different parts of the central nervous system.

### *Bdnf* mRNA levels in adult brain in different mouse strains

Next, we aimed to determine whether *Bdnf* levels are similar in different laboratory mouse strains, and we found that the levels of *Bdnf* in the whole brain and hippocampi of adult mice are relatively stable between the strains (Supplementary Figure S1A, Supplementary Table 4). The proportions of different *Bdnf* transcripts are roughly consistent between different mouse strains, although the hippocampus of PWD/Ph strain exhibits higher proportion of exon I and IIc-containing transcripts than the other analyzed strains (Supplementary Figure S1A). Despite some fluctuations, total *Bdnf* mRNA levels and proportions of transcripts are similar between mouse strains.

### *Bdnf* mRNA levels in different brain regions of adult mouse and rat

A comprehensive analysis of different brain regions in adult mouse shows that total *Bdnf* mRNA levels vary substantially, with the highest levels of *Bdnf* mRNA found in the hippocampus and different cortical regions, and the lowest in the striatum (caudate putamen; Supplementary Figure S2A; Supplementary Table 5). *Bdnf* exon I-, IIc-, IV- and VI-containing transcripts are the major *Bdnf* transcripts and are expressed in different brain regions, with *Bdnf* exon I and IV generally accounting for over half of the *Bdnf* transcripts (Supplementary Figure S2A). Interestingly, the proportion of exon I and IIc-containing transcripts is the highest in the septum, amygdala, thalamus, hypothalamus, midbrain, and pons and medulla, where these transcripts form approximately half of the total *Bdnf* mRNA pool (Supplementary Figure S2A). In contrast, retina, pituitary gland, and cerebellum almost completely lack *Bdnf* exon I-containing transcripts (Supplementary Figure S2A).

Similarly, in the adult rat central nervous system the highest total *Bdnf* mRNA levels are in the hippocampus and different cortical regions (Supplementary Figure S3A; Supplementary Table 6). Major *Bdnf* transcripts contain exons I, IIc, IV, or VI, however, exon III also remarkably contributes to the total *Bdnf* pool in many brain regions (Supplementary Figure S3A). It is worth noting that in this dataset, *Bdnf* exon VI-containing transcripts do not contribute significantly to total *Bdnf* mRNA levels in different cortical regions (Supplementary Figure S3A). Altogether, our results show that total *Bdnf* mRNA levels and proportion of transcripts vary between brain regions in both adult mouse and rat.

### *TrkB* mRNA expression levels in mouse and rat brain during development

Next, we aimed to revisit the expression of different *Ntrk2* mRNAs encoding different TrkB protein isoforms (hereafter referred to as *TrkB* mRNAs). Our analysis shows that both full-length *TrkB* (hereafter *TrkB-FL*) and truncated *TrkB-T1* mRNA levels increase remarkably in both mouse (Figure 1C) and rat (Figure 1D) whole brain during development. In mouse the mRNA levels of *TrkB-T1* and *TrkB-FL* increase and are mostly similar throughout development, except in the hypothalamus and adult mouse cerebellum where the *TrkB-T1* mRNA levels are much higher than the levels of *TrkB-FL* (Figure 1C). Interestingly, in rat the expression levels of *TrkB-T1* isoform reach higher levels than the full-length isoform in both cerebral cortex and cerebellum during development (Figure 1D). Notably, the mRNA levels of *TrkB-Shc* isoform are almost undetectable in the studied mouse and rat brain regions (Figures 1C,D). In different adult mouse and rat brain regions, *TrkB-T1* mRNA levels are consistently higher than full-length *TrkB* isoforms (Supplementary Figures S2B, S3B). Collectively, our results show upregulation of *TrkB* isoform mRNA levels during murine development, with higher levels of *TrkB-T1* than *TrkB-FL*.

The mRNA levels of different *TrkB* isoforms vary in different mouse strains. For example, the mRNA levels of *TrkB-T1* and *TrkB-FL* isoforms are roughly equal in the whole brain of adult BALB/cJ and C57BL6/6NJ mice, whereas *TrkB-T1* is expressed at a higher level than *TrkB-FL* in adult NZO, NOD/Ltj, and 129S1 mice (Supplementary Figure S1B). The mRNA levels of different *TrkB* isoforms are more stable in the hippocampus of different mouse strains (Supplementary Figure S1B). In summary, our results reveal that BDNF signaling may differ between mouse strains due to the varying expression of *TrkB* isoforms.

### *p75NTR* mRNA expression levels in mouse and rat brain during development

Next, we analyzed the expression levels of the other BDNF receptor, p75NTR. Our results show that the expression of the *Ngfr* gene (encoding p75NTR, hereafter referred to as *p75NTR* mRNA) mainly decreases in both mouse (Figure 1E) and rat (Figure 1F) development, implying that TrkB, whose expression shows the opposite trend, is the main receptor of BDNF in the adult brain. The mRNA levels of *p75NTR* are low and slightly differ between mouse strains in the whole brain but are more similar in the hippocampi (Supplementary Figure S1C). Interestingly, in the adult mouse brain the levels of *p75NTR* are generally very low, with the highest expression seen in the retina (Supplementary Figure S2C), where *TrkB* isoform mRNAs are expressed at very low levels (Supplementary Figure S2B). In adult rat brain regions, the levels of *p75NTR* are also low, with the highest levels detected in the medial preoptic area (Supplementary Figure S3C). Taken together, our results show a developmental decrease in the levels of *p75NTR* in the murine central nervous system.

### Expression levels of total *Bdnf* mRNA and different transcripts in mouse and rat non-neural tissues during development

**Figure 2:**
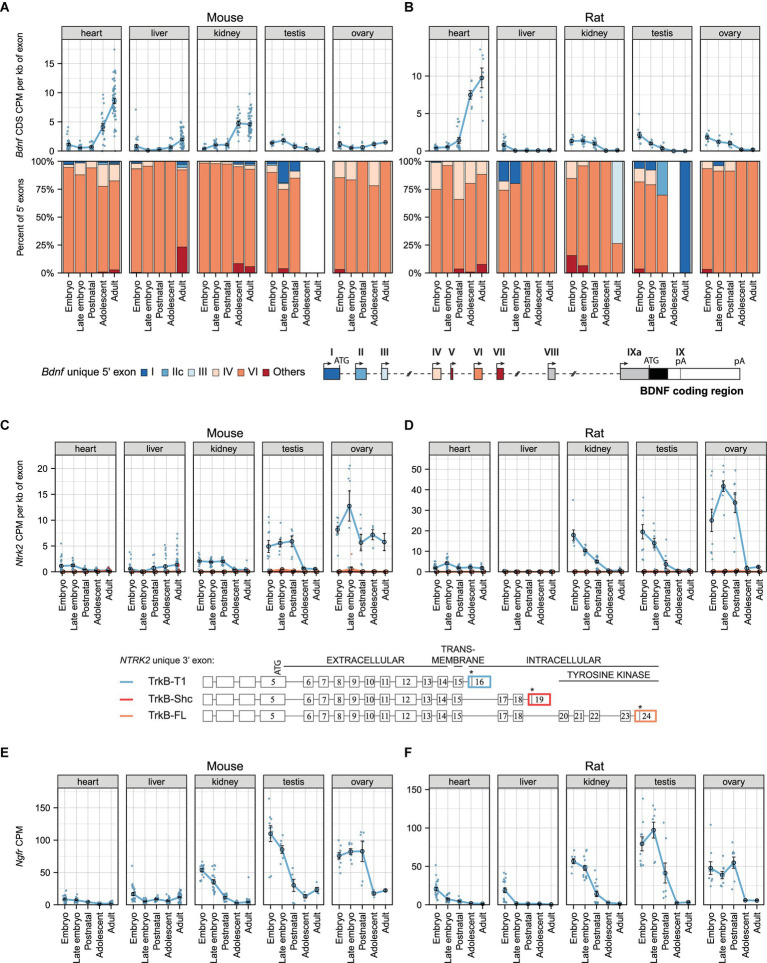
The expression levels of mRNAs encoding BDNF and its receptors TrkB (*Ntrk2* mRNAs) and p75NTR (*Ngfr* mRNAs) during development in murine non-neural tissues. Meta-analysis of *Bdnf*, *Ntrk2* (encoding TrkB) and *Ngfr* (encoding p75NTR) expression in mouse **(A,C,E)** and rat **(B,D,F)** non-neural tissues during development. The expression levels in embryo (E10.5-E14.5/E11-E15 mouse/rat), late embryo (E15.5-E18.5/E17-E20 mouse/rat), postnatal (P0-P14), adolescent (P22-56) and adult (P62+) animals are shown. **(A,B)** Total *Bdnf* expression measured by levels of *Bdnf* coding sequence (CDS, upper panel) and distribution of levels of *Bdnf* 5′ exons (lower panel) are shown as depicted on the schematics of murine gene structure. The exons indicated with gray color were not included in the analysis of 5′ exons. White box in the proportion of *Bdnf* 5′ exons indicates that the expression of *Bdnf* was too low for this calculation. **(C,D)** The mRNA levels of different *TrkB* isoforms based on the levels of unique 3′ exons of the *Ntrk2* gene (rat or mouse counterpart of human exon 16, 19, 24). The 3′ exons specific for *TrkB* isoforms *TrkB-T1*, *TrkB-Shc,* and *TrkB-FL* are shown on the schematics (adapted from Luberg et al. (2010)). Asterisks on the schematics mark stop-codons. **(E,F)** Total *Ngfr* mRNA expression levels. Data from individual animals are shown as small dots, circles indicate mean values and error bars represent standard error of the mean (SEM). All used datasets and underlying data are shown in Supplementary Table 7. CDS – coding sequence, CPM – counts per million.

We next focused on describing the expression of *Bdnf* and its receptors in non-neural tissues. Our analysis shows that during murine development the total *Bdnf* mRNA levels increase in mouse heart and decrease in testis (Figure 2A; Supplementary Table 7), whereas in rat total *Bdnf* mRNA levels strongly increase in the heart, but decrease in the liver, kidney, testis, and ovary to almost undetectable levels by adulthood (Figure 2B). In contrast to mouse, *Bdnf* mRNA levels in rat kidney decrease during development (Figure 2B).

We next focused on the expression of different *Bdnf* 5′ transcripts and determined that *Bdnf* exon VI-containing transcripts are the major *Bdnf* transcripts in murine non-neural tissues, contributing to over 75% of total *Bdnf* transcripts throughout development (Figures 2A,B). Interestingly, *Bdnf* exon IV transcripts are expressed, although at low levels, in both mouse and rat heart (Figures 2A,B). Also, *Bdnf* exon I and IIc-containing transcripts are present in the testis during early development in both mouse and rat (Figures 2A,B). Collectively, our results show developmentally regulated levels of *Bdnf* mRNA in certain murine non-neural tissues where the majority of *Bdnf* transcripts contain exon VI.

### mRNA levels of *TrkB* isoforms and *p75NTR* in mouse and rat non-neural tissues during development

We next investigated mRNA levels of different *TrkB* isoforms during the development of mouse and rat in non-neural tissues. The major *TrkB* isoform in murine non-neural tissues is *TrkB-T1*, while *TrkB-FL* and *TrkB-Shc* isoforms are almost undetectable (Figures 2C,D). In the kidney, testis, and ovary, the expression of *TrkB-T1* isoform is high in early developmental stages and decreases during development (Figures 2C,D). Our analysis also shows that *TrkB-T1* isoform is expressed at very low levels in both mouse and rat heart and liver (Figures 2C,D). Similar expression patterns are observed for *p75NTR* mRNA levels (Figures 2E,F). Altogether, our results show developmental downregulation of *TrkB-T1* isoform and *p75NTR* in murine non-neural tissues.

### *Bdnf*, *TrkB*, and *p75NTR* mRNA levels in various tissues in adult murines

We next conducted a meta-analysis of adult mouse and rat neural and non-neural tissues, combining data from various sources (see Supplementary Table 8 for details). Compared to non-neural tissues, *Bdnf* expression levels are consistently higher in the murine brain (Supplementary Figures S4A,B; Supplementary Table 8). Among the non-neural tissues that significantly express *Bdnf*, the heart and lung show similar *Bdnf* mRNA levels to those found in the brain. Other non-neural tissues that express *Bdnf* include the stomach, kidney, and heart in mouse (Supplementary Figure S4A) and the esophagus in rat (Supplementary Figure S4B). While the dominant *Bdnf* transcripts in both mouse and rat brain contain exons I, IIc, IV and VI, in most non-neural tissues the major *Bdnf* transcripts contain exon VI (Supplementary Figures S4A,B). Transcripts containing *Bdnf* exons I, IIc, and IV also contribute to total *Bdnf* mRNA levels in adult mouse skin, bone, and adipose tissue (Supplementary Figure S4A). In summary, our results show that some adult murine non-neural tissues express total *Bdnf* mRNA at comparable levels to those in the brain and the majority of *Bdnf* transcripts contain exon VI.

Interestingly, while both *TrkB-FL* and *TrkB-T1* isoforms are expressed in adult murine brain, only *TrkB-T1* mRNA is expressed in the non-neural tissues – in mouse lung, skin, adipose tissues, esophagus, ovary, and adrenal gland (Supplementary Figure S4C) and in rat lung, esophagus, ileum, and spleen (Supplementary Figure S4D). *p75NTR* mRNA levels are expressed highest in mouse in skin, mesenteric adipose tissue, spleen, ovary, and testis, and the levels in other tissues show similar low expression levels as in the brain (Supplementary Figure S4E). In rat the *p75NTR* mRNA levels are highest in the cerebellum, thymus, spleen, and various parts of the digestive system (Supplementary Figure S4F). Overall, our results show *TrkB-T1* and *p75NTR* expression in some adult murine non-neural tissues.

### *Bdnf*, *TrkB*, and *p75NTR* mRNA levels in various mouse tissues during aging

Finally, we analyzed *Bdnf*, *TrkB*, and *p75NTR* mRNA levels in aging mouse. In the whole brain and most studied mouse non-neural tissues, total *Bdnf* mRNA levels are relatively stable throughout adult life, except for gonadal adipose tissue and lung, where the total *Bdnf* mRNA levels increase during aging (Supplementary Figure S5A; Supplementary Table 9). In non-neural tissues, *Bdnf* transcripts mainly contain exon VI, albeit in heart a very consistent expression of both *Bdnf* exon IV- and VI-containing transcripts is observed (Supplementary Figure S5A). During mouse aging the proportion of *Bdnf* exon I- and IIc-containing transcripts increases in mesenteric adipose tissue and decrease in gonadal adipose tissues (Supplementary Figure S5A). In the brain, *TrkB-T1* and *TrkB-FL* isoforms are both stably expressed throughout life (Supplementary Figure S5B). Of the analyzed non-neural tissues, skin, adipose tissues, and lung show expression of *TrkB-T1* isoform, where no major changes in the expression during aging are seen (Supplementary Figure S5B). *p75NTR* is expressed in the skin, different adipose tissues, liver, and spleen, with the expression levels increasing in the skin and spleen and decreasing in the mesenteric adipose tissues during aging (Supplementary Figure S5C). Collectively, *Bdnf* and *TrkB* mRNA levels do not change notably within one tissue during mouse aging.

### *BDNF*, *TRKB*, and *P75NTR* mRNA expression levels in human brain during development

**Figure 3:**
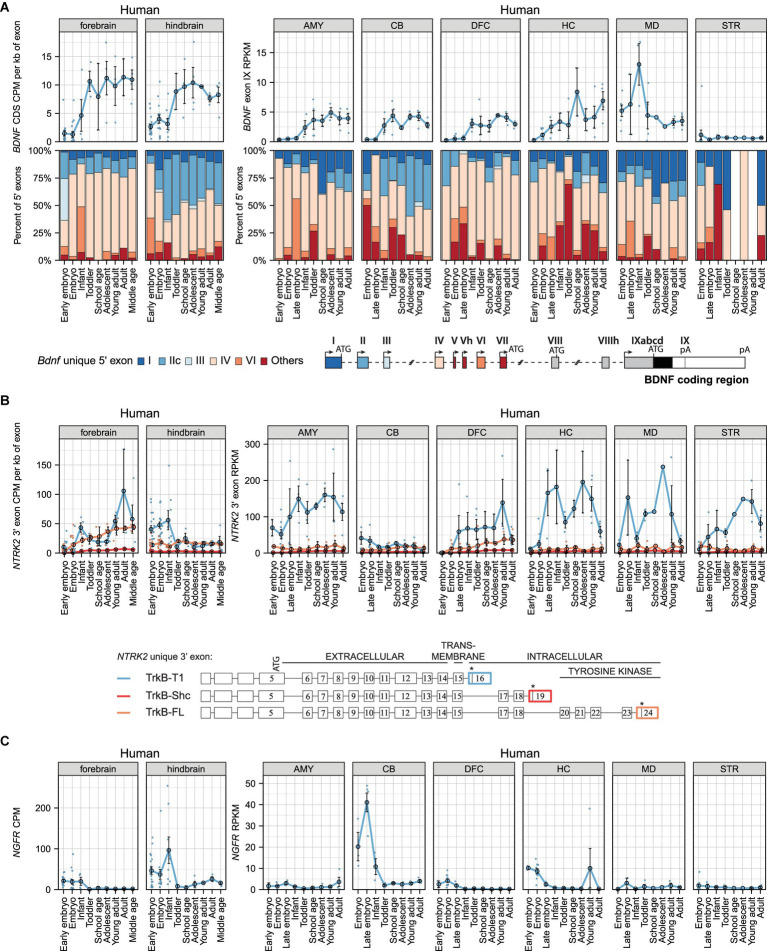
The expression levels of mRNAs encoding BDNF and its receptors TRKB (*NTRK2* mRNAs) and p75NTR (*NGFR* mRNAs) in human brain regions throughout development. Visualization of *BDNF*, *NTRK2* (encoding TRKB) and *NGFR* (encoding P75NTR) expression data from Cardoso-Moreira et al. (2019) (left panels) and human developmental transcriptome data from BrainSpan project (right panels). The expression levels in early embryo [0–9 postcoital week (PCW)], embryo (10–19 PCW), late embryo (20–39 PCW), infant (younger than 12 months), toddler (1–4 years old), school age (7–8 years old), adolescent (10–17 years old), young adult (18–29.99 years old), adult (30–39.99 years old), and middle-aged (40–58 years old) humans are shown. **(A)** Total *BDNF* expression measured by levels of *BDNF* coding sequence (CDS, upper panel) and distribution of levels of *BDNF* 5′ exons (lower panel) are shown as depicted on the schematics of human gene structure. The exons indicated with gray color were not included in the analysis of 5′ exons. **(B)** The mRNA levels of different TrkB isoforms based on the levels of unique 3′ exons of *NTRK2* gene (exon 16, 19, 24). The 3′ exons specific for *TRKB* isoforms *TRKB-T1*, *TRKB-SHC*, and *TRKB-FL* are shown on the scheme (adapted from Luberg et al. (2010)). Asterisks on the schematics mark stop-codons. **(C)** Total *NGFR* mRNA expression levels. Data from individual animals are shown as small dots, circles indicate mean values and error bars represent standard error of the mean (SEM). All used datasets and underlying data are shown in Supplementary Table 10. AMY – amygdaloid complex, CB – cerebellar cortex, DFC – dorsolateral prefrontal cortex, HC – hippocampus (hippocampal formation), MD – mediodorsal nucleus of thalamus, STR – striatum, CDS – coding sequence, CPM – counts per million.

Next, we aimed to determine the expression pattern of *BDNF* and its receptors in humans. Compared to the murine *BDNF* gene structure, the human *BDNF* gene is more complex and has two additional 5′ exons (Vh and VIIIh) (Pruunsild et al., 2007). Overall, *BDNF* transcripts are generated similarly in humans and murines, however, in rare cases multiple 5′ exons are used in humans (e.g., *BDNF* exons VIII and VIIIh can be used as internal exons) (Pruunsild et al., 2007). Our analysis shows that total *BDNF* mRNA levels rise in different brain regions during human development (Figure 3A; Supplementary Table 10), reaching the highest levels in the adult hippocampus and infant thalamus (Figure 3A). In the striatum almost no *BDNF* mRNA can be detected (Figure 3A). Taken together, our results show developmental upregulation of *BDNF* mRNA levels in the human brain.

Our analysis further shows that *BDNF* exon IV-containing transcripts are the most ubiquitously expressed in different brain regions, while the proportions of *BDNF* exon VI-containing transcripts are low (Figure 3A). Notably, in the hindbrain and cerebellum *BDNF* exon IIc transcripts comprise a remarkable proportion of total *BDNF* mRNA, and this proportion increases during development, whereas the proportion of *BDNF* exon I transcripts is very low in these brain regions (Figure 3A). In contrast, in the mediodorsal nucleus of thalamus, *BDNF* exon I-containing transcripts notably contribute to the total *BDNF* mRNA pool throughout development and a high proportion of exon I transcripts is also seen in the amygdala and adult hippocampus (Figure 3A). Overall, it appears that *BDNF* exon IV-containing transcripts are the main transcripts in most human brain regions (except the cerebellum), whereas other transcripts show brain region-specific expression patterns.

Next, we analyzed the mRNA levels of *TRKB* and *P75NTR*. Our analysis shows that *TRKB-T1* is the main isoform expressed in most brain regions (except the cerebellum) across all developmental stages (Figure 3B). Furthermore, *TRKB-T1* mRNA levels remarkably decrease in the adult hippocampus, thalamus, and striatum compared to levels in young adults (Figure 3B). Interestingly, the mRNA levels of *TRKB-FL* isoform increase in the forebrain and dorsolateral prefrontal cortex during development (Figure 3B). In other studied brain regions, the mRNA levels of *TRKB-FL* are low and *TRKB-SHC* expression is almost undetectable (Figure 3B). Finally, we focused on *P75NTR* mRNA levels in the human brain and determined that it is expressed at low levels, except in the embryonic hindbrain and cerebellum, which showed high expression levels (Figure 3C). Our results suggest that in adult humans the main BDNF receptor in the brain is TRKB.

### *BDNF*, *TRKB*, and *P75NTR* mRNA levels in human non-neural tissues during development

**Figure 4:**
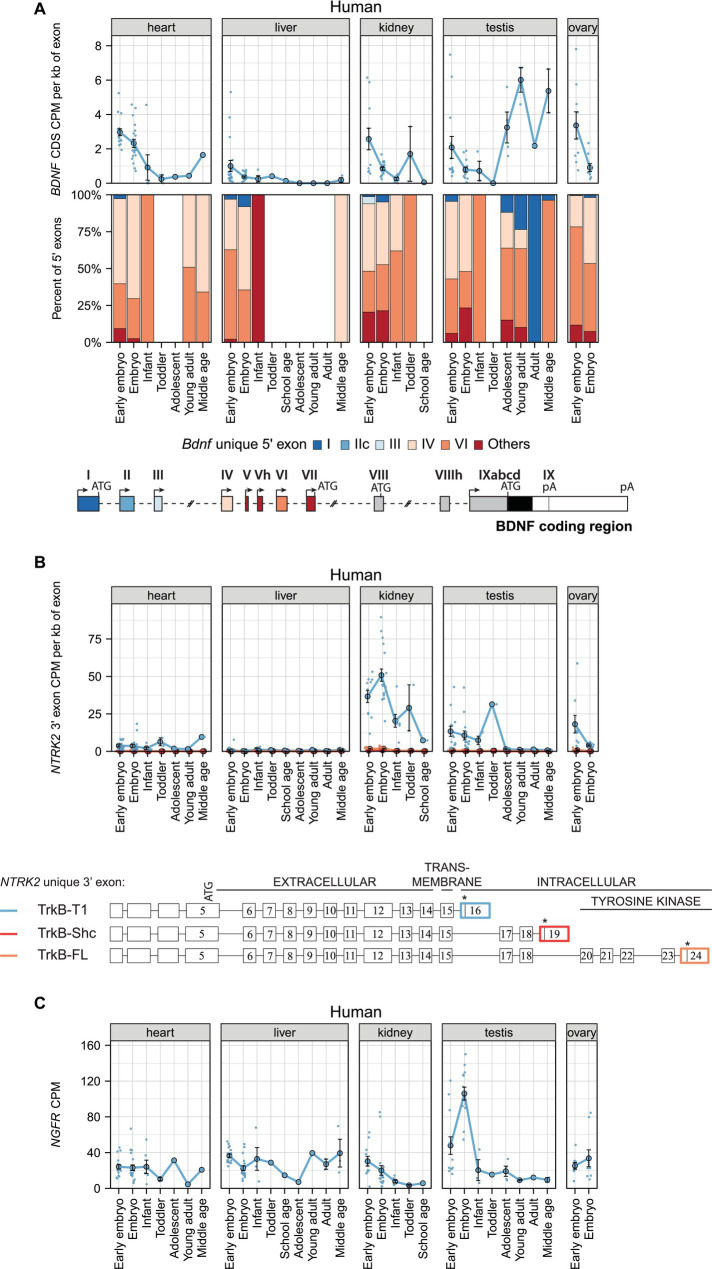
The expression levels of mRNAs encoding BDNF and its receptors TRKB (*NTRK2* mRNAs) and p75NTR (*NGFR* mRNAs) in human non-neural tissues. The expression levels of *BDNF*, *NTRK2* (encoding TRKB), and *NGFR* (encoding P75NTR) mRNAs in early embryo [0–9 postcoital week (PCW), embryo (10–19 PCW), late embryo (20–39 PCW), infant (younger than 12 months), toddler (1–4 years old), school age (7–8 years old), adolescent (10–17 years old), young adult (18–29.99 years old), adult (30–39.99 years old), and middle-aged (40–58 years old) humans are shown (data from Cardoso-Moreira et al. (2019)]. **(A)** Total *BDNF* mRNA levels measured by levels of *BDNF* coding sequence (CDS, upper panel) and distribution of levels of *BDNF* 5′ exons FIGURE 4 (Continued)(lower panel) are shown as depicted on the schematics of human *BDNF* gene structure. The exons indicated with gray color were not included in the analysis of 5′ exons. **(B)** The mRNA levels of different *TRKB* isoforms based on the levels of unique 3′ exons of *NTRK2* gene (exon 16, 19, 24). The 3′ exons specific for *TRKB* isoforms *TRKB-T1*, *TRKB-SHC*, and *TRKB-FL* are shown on the scheme (adapted from Luberg et al. (2010)). Asterisks on the scheme mark stop-codons. **(C)** Total *NGFR* mRNA expression levels. All used datasets and underlying data are shown in Supplementary Table 11. Data from individual animals are shown as small dots, circles indicate mean values and error bars represent standard error of the mean (SEM). CDS – coding sequence, CPM – counts per million.

Next, we investigated *BDNF* expression in human non-neural tissues. Our analysis shows that *BDNF* is expressed at low levels and its expression decreases in the heart, kidney, and ovary, and increases in the testis during development (Figure 4A; Supplementary Table 11). In adult human, *BDNF* mRNA expression is highest in the nervous system, but also in the arteries and heart, prostate, and lung (Supplementary Figure S6A; Supplementary Table 12). *BDNF* exon IV and VI-containing transcripts are the main *BDNF* transcripts expressed in human non-neural tissues (Figure 4A; Supplementary Figure S6A).

The BDNF receptor *TRKB-T1* is the only TRKB isoform that is expressed in human non-neural tissues, with the highest levels in embryonic kidney, but found at lower levels also in the heart, testis, and ovary (Figure 4B). During development, the levels of *TRKB-T1* decrease in the kidney, testis, and ovary (Figure 4B). Interestingly, in adult human *TRKB-T1* is also expressed in non-neural tissues where *BDNF* is expressed, e.g., in arteries, mammary tissue, adipose tissue, and thyroid (Supplementary Figure S6B). Finally, *P75NTR* is expressed in the heart, liver, and ovary at similar and stable levels throughout development, while the expression of *P75NTR* decreases during development in the kidney and testis (Figure 4C). In adult human, the mRNA expression of *P75NTR* is notably higher in non-neural tissues than in different brain regions, and surprisingly shows the highest expression in the tibial nerve (Supplementary Figure S6C).

### Evolutionary analysis of *BDNF* gene expression in mammalian development

**Figure 5:**
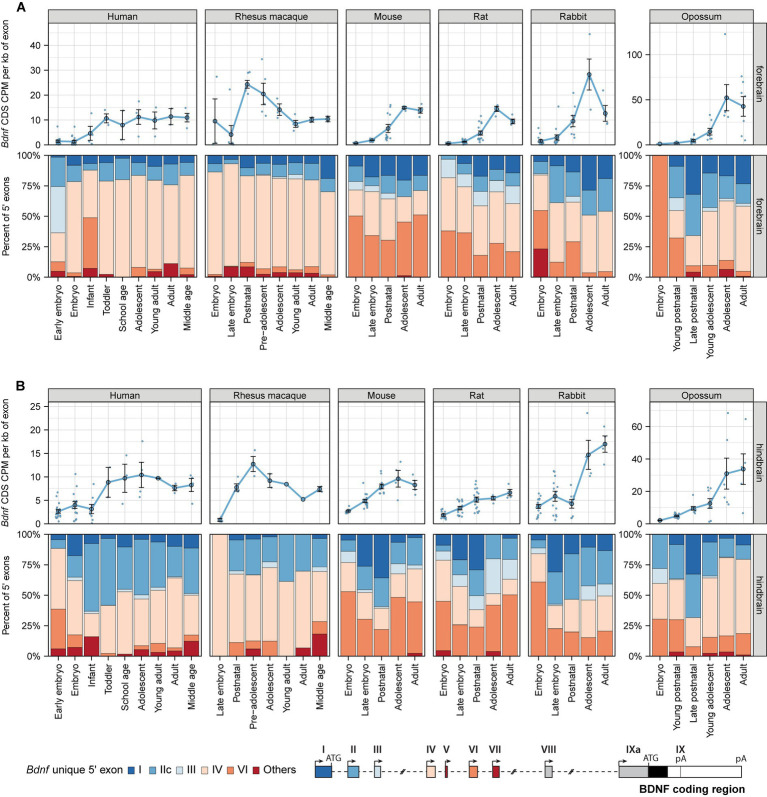
*BDNF* mRNA expression levels in the brain of different mammals. Total *BDNF* mRNA levels in forebrain **(A)** and hindbrain **(B)** measured by levels of *BDNF* coding sequence (CDS, upper panels) and distribution of levels of *BDNF* 5′ exon-specific mRNAs (lower panels) are shown with the exons depicted below in the schematics of murine *Bdnf* gene structure. The exons indicated with gray color were not included in the analysis of expressed 5′ exons. *BDNF* mRNA levels in humans are shown in early embryo (0–9 postcoital week (PCW)), embryo (10–19 PCW), late embryo (20–39 PCW), infant (younger than 12 months), toddler (1–4 years old), school age (7–8 years old), adolescent (10–17 years old), young adult (18–29.99 years old), adult (30–39.99 years old), and middle-aged (40–58 years old); in rhesus macaques in embryo (E93-E109), late embryo (PE112-E130), postnatal (P0-P24), pre-adolescent (0.5–1 years old), adolescent (2–3 years old), young adult (8–11 years old), adult (14–15 years old), and middle-aged (20–26 years old) animals; in mouse and rats in embryo (E10.5-E14.5/E11-E15 mouse/rat), late embryo (E15.5-E18.5/E17-E20 mouse/rat), postnatal (P0-P14), adolescent (P22-56), and adult (P62+) animals; in rabbits in embryo (E12-E19.5), late embryo (E21-E27), postnatal (P10-P14), adolescent (P84), and adult (P186-P548) animals; in opossums in late embryo (E13.5), young postnatal (P0-P6), late postnatal (P10-P21), young adolescent (P28-P60), adolescent (P90-P120), and adult (P150-P180) animals [data from (2019)]. All used datasets and underlying data are shown in Supplementary Table 13. Data from individual animals are shown as small dots, circles indicate mean values and error bars represent standard error of the mean (SEM). CDS – coding sequence, CPM – counts per million.

Next, to study whether the expression of *BDNF* is evolutionarily conserved among mammals, we focused on *BDNF* mRNA levels and proportions of its transcripts throughout mammalian development. Our results show that during development total *BDNF* mRNA levels generally increase and reach similar levels in human, rhesus macaque, mouse, rat, and rabbit forebrain and hindbrain, but reach remarkably higher levels in the adult opossum forebrain and hindbrain (note different scale for opossum in the figure) (Figure 5; Supplementary Table 13). Although total *BDNF* mRNA levels are quite similar in the forebrain of different mammals, we noted significant differences in the expression of different *BDNF* transcripts (Figure 5A). In human and rhesus macaque forebrain, vast majority (> 75%) of *BDNF* transcripts are exon IV mRNAs, followed by lower levels of exon I and IIc mRNAs and almost no contribution by exon VI mRNAs (Figure 5A). Surprisingly, in mouse and rat forebrain, all *Bdnf* 5′ exons contribute to *Bdnf* mRNA levels (Figure 5A). Although exon VI mRNAs are also prevalent in the early development of rabbit and opossum forebrain, the proportion of exon VI-containing transcripts drops quickly during development to almost undetectable levels in the adult organism. It appears that the proportion of transcripts arising from the first cluster of *BDNF* exons (exon I, IIc, and III) has decreased during mammalian evolution, with the highest levels seen in opossum and lowest in primates (Figure 5A). In human and rhesus macaque hindbrain, *BDNF* transcripts containing exon IIc and IV are the main transcripts, while in mouse, rat, rabbit, and opossum *Bdnf* exon VI also contributes notably to the total pool of *BDNF* (Figure 5B). Collectively, our results show evolutionary differences in the expression of different *BDNF* transcripts in mammalian brain.

Next, we studied *BDNF* mRNA levels in non-neural tissues during mammalian development. Total *BDNF* mRNA levels are mostly similar within one tissue among the different studied mammals (Supplementary Figure S7; Supplementary Table 14). Some exceptions are rhesus macaque heart, mouse and rabbit kidney, and opossum and rhesus macaque testis, where *BDNF* mRNA levels are higher than in the respective tissue in other studied mammals (Supplementary Figures S7A,C,D, respectively). Overall, the majority of *BDNF* transcripts contain exon VI, however, *BDNF* exon IV also contributes to *BDNF* mRNA levels in these mammals, although at a much lower level in murines when compared to the other studied mammals (Supplementary Figure S7).

### Expression of *BDNF* transcripts with alternative 3’ UTRs

In addition to different promoter regions and the resulting 5′ exons in *BDNF* gene, *BDNF* exon IX contains alternative polyadenylation sites that can result in transcripts with either a short or long 3′ untranslated region (UTR) (Timmusk et al., 1993b; Fukuchi and Tsuda, 2010). *BDNF* transcripts with short 3’ UTR are more stable (Castrén et al., 1998) and are more associated with polysomes than transcripts with long 3’ UTR, suggesting better translatability (Timmusk et al., 1994b). As there are functional differences between the transcripts with different 3’ UTR lengths (Lekk et al., 2023), we set out to analyze the expression levels of *BDNF* transcripts with different 3’ UTRs in different tissues and to see whether the choice of the polyadenylation signal is evolutionarily conserved.

**Figure 6:**
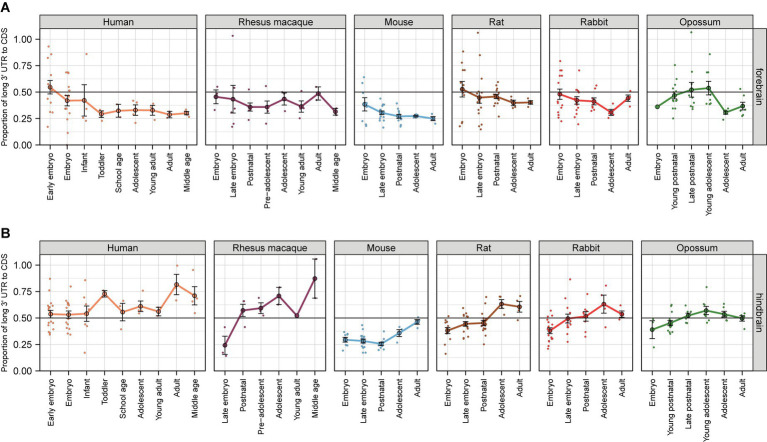
The proportion of *BDNF* transcripts with long 3′ untranslated region in the brain of different mammals. The ratio of *BDNF* transcripts with long 3′ untranslated region (UTR) is shown in forebrain **(A)** and hindbrain **(B)** relative to total *BDNF* mRNA levels measured by levels of *BDNF* coding sequence (CDS). All used datasets and underlying data are shown in Supplementary Table 15. For more information on age classifications, see legend of Figure 5 (data from Cardoso-Moreira et al., 2019). Data from individual animals are shown as small dots, circles indicate mean values and error bars represent standard error of the mean (SEM). CDS – coding sequence.

Our analysis shows that in the forebrain of human, rhesus macaque, mouse, rat, rabbit, and opossum the relative levels of *BDNF* transcripts with long 3’ UTR slightly decrease during development, dropping down to 30% of total *BDNF* transcripts in adulthood (Figure 6A; Supplementary Table 15). In contrast, in hindbrain the relative levels of transcripts with long 3’ UTR slightly increase during development, reaching up to 50% of all *Bdnf* transcripts in adult animals, and the highest increase is observed in middle aged rhesus macaques (up to 80%) (Figure 6B). In non-neural tissues, the relative levels of *BDNF* transcripts with long 3’ UTR is either stable with around ~50% of all transcripts or slightly decrease during development in the heart, liver, kidney, and ovary, and substantially decrease in the testis of the studied animals (except in rabbit) (Supplementary Figure S8; Supplementary Table 16). Collectively, our analysis indicates that the usage of *BDNF* alternative polyadenylation signals is relatively conserved in mammalian evolution.

Next, we conducted a comprehensive adult meta-analysis of different datasets to further describe *BDNF* transcripts with alternative 3’ UTRs in several different tissues in adult human, mouse, and rat. In agreement with our previous results, the highest proportion of long 3’ UTR transcripts is observed in human hindbrain (Supplementary Figure S9A; Supplementary Table 17), mouse cerebellum, bone and bone marrow, spleen, small intestine, and ovary (Supplementary Figure S9B), and rat cerebellum, uterus, adrenal gland, and lung (Supplementary Figure S9C). Altogether, our data suggests differential regulation of *BDNF* alternative polyadenylation in different tissues.

### *BDNF* expression in different cell types based on single cell RNA sequencing

**Figure 7:**
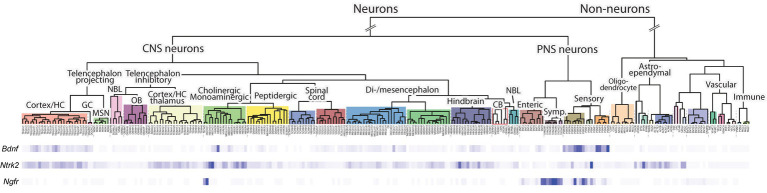
The expression of *Bdnf*, TrkB (*Ntrk2* gene), and p75NTR (*Ngfr* gene) mRNA in adolescent mouse nervous systems according to single cell RNA sequencing. Single cell RNA sequencing data of postnatal day 10–30 mice from Zeisel et al. (2018) was visualized using Mouse Brain Atlas from the Linnarson lab (http://mousebrain.org/). A deeper blue color indicates stronger expression of the indicated gene. The different cell types and their origins are shown above.

We next used publicly available single cell RNA-sequencing data accessible through the CellxGene database (Chan Zuckerberg Initiative, n.d.) to describe the cell types where *BDNF* is expressed. As expected, in the brain the most prevalent expression of *BDNF* is in glutamatergic neurons in both mouse (Table 1) and human (Table 2). Unfortunately, the studied CellxGene single cell datasets did not contain any data on sensory neurons, where the function of BDNF protein was discovered (Barde et al., 1978, 1980, 1982) and which are substantially lost in *Bdnf* knock-out animals (Ernfors et al., 1994; Jones et al., 1994). To further elucidate the expression of *Bdnf*, *TrkB* and *p75NTR* expression in the nervous system, we used single cell RNA sequencing data of the adolescent mouse nervous system by Zeisel et al. (2018) (Web tool accessible from http://mousebrain.org/adolescent/). According to this data, the *Bdnf* gene is mainly expressed in excitatory neurons in the central nervous system, and in sensory neurons in the peripheral nervous system (Figure 7). In contrast, *TrkB* shows widespread expression in different neurons throughout the nervous system, as well as in astrocytes (Figure 7). The expression of *p75NTR* is mainly constrained to certain subtypes of cholinergic neurons, and sympathetic and sensory neurons (Figure 7).

**Table 1:** *Bdnf* mRNA expression in mouse tissues according to single cell RNA sequencing.

Tissue	Cell type	BDNF detected in
Brain	Glutamatergic neuron	16.96%
	neuron	2.71%
	GABAergic neuron	0.50%
Urinary bladder	Bladder cell	7.19%
Musculature	Smooth muscle cell	6.87%
	Skeletal muscle satellite cell	0.97%
	Mesenchymal stem cell	0.56%
Exocrine gland	Basal cell	4.47%
	Luminal epithelial cell of mammary gland	1.94%
Lung	Mesothelial cell of visceral pleura	4.15%
	Vascular associated smooth muscle cell	2.76%
	Type I pneumocyte	2.57%
	Mesenchymal cell	0.53%
Heart	Fibroblast of cardiac tissue	1.66%
Kidney	Kidney proximal convoluted tubule epithelial cell	0.61%

Only cell types with at least 500 identified cells and *Bdnf* expression in at least 0.5% of the cells are shown (CellxGene database).

**Table 2:** *BDNF* mRNA expression in human tissues according to single cell RNA sequencing.

Tissue	Cell type	BDNF detected in
Lung	Mesothelial cell	14.13%
	Type I pneumocyte	3.09%
	Muscle cell	0.78%
	Epithelial cell	0.58%
Spleen	Vascular associated smooth muscle cell	9.17%
	Megakaryocyte	1.23%
Bladder organ	myofibroblast cell	6.02%
	pericyte	0.69%
Brain	L2/3–6 intratelencephalic projecting glutamatergic cortical neuron	5.75%
	L5 extratelencephalic projecting glutamatergic cortical neuron	1.39%
	Corticothalamic-projecting glutamatergic cortical neuron	1.37%
	Neuron	0.58%
Vasculature	Smooth muscle cell	4.23%
	Pericyte	0.60%
Small intestine	Pericyte	4.10%
	Fibroblast	0.67%
Colon	Pericyte	3.82%
	Mesothelial cell	2.95%
Heart	Cardiac muscle myoblast	3.76%
	Regular ventricular cardiac myocyte	1.85%
	Hepatocyte	1.20%
	Native cell	1.04%
Eye	Retinal ganglion cell	2.84%
	Retinal bipolar neuron	1.19%
Endocrine gland	Epithelial cell of thymus	2.58%
Skin of body	Keratinocyte	2.23%
Liver	Megakaryocyte	2.11%
	Epithelial cell	1.54%
Bone marrow	Megakaryocyte	1.86%
	Osteoblast	1.01%
	Preosteoblast	0.96%
	Plasma cell	0.58%
Axilla	Epithelial cell	1.48%
Tongue	Keratinocyte	0.93%
	Basal cell	0.59%
Pleural fluid	Epithelial cell	0.72%
Digestive system	Vascular associated smooth muscle cell	0.67%
Ovary	Granulosa cell	0.56%
	Theca cell	0.52%
Respiratory system	Epithelial cell	0.53%

Only cell types with at least 500 identified cells and *BDNF* expression in at least 0.5% of the cells are shown (CellxGene database).

In mouse non-neural tissues, *Bdnf* is expressed in luminal epithelial cells in the mammary gland, in cardiac fibroblasts, in various cell types in the lung, in smooth muscle cells and in bladder cells (Table 1). In human non-neural tissues, *BDNF* is mainly expressed in epithelial and mesothelial cells, e.g., in the colon and lung (Table 2). In addition, we determined widespread *BDNF* gene expression in megakaryocytes, pericytes and smooth muscle cells of the vasculature (Table 2). In the human heart, *BDNF* is expressed in both myocytes and myoblasts (Table 2). In summary, our data show that only certain types of specialized cells produce *BDNF*.

### BDNF protein levels in mouse and rat brain during development

*BDNF* mRNA and protein expression levels do not always spatially correlate, as BDNF protein can be anterogradely transported between brain regions (Conner et al., 1997). For example, while there is almost no *Bdnf* mRNA in the striatum (Hofer et al., 1990; Timmusk et al., 1994b), the existing BDNF protein (Radka et al., 1996; Baquet et al., 2004) is transported there from the cerebral cortex (Altar et al., 1997; Baquet et al., 2004) and is necessary for the proper development of the striatum (Baquet et al., 2004). Moreover, there are high BDNF protein levels in the adult rat pituitary gland although *Bdnf* mRNA levels there are low (Yan et al., 1997). Therefore, it is possible that BDNF protein can be detected in tissues lacking *BDNF* mRNA. To investigate BDNF protein levels, we analyzed different brain regions throughout the development of the commonly used mouse strains BALB/c and C57BL/6 J and Wistar rat by Western blot analysis.

**Figure 8:**
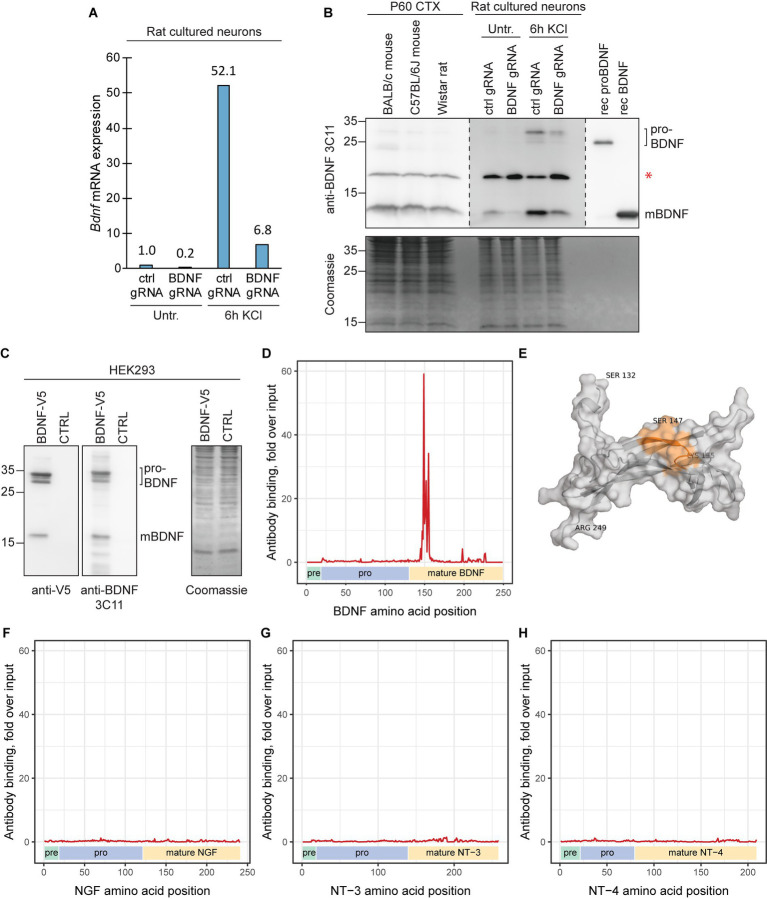
The epitope of Icosagen 3C11 anti-BDNF antibody resides between BDNF amino acids 147–155. **(A)** The specificity of the anti-BDNF 3C11 antibody (Icosagen) was tested in cultured rat cortical neurons. *Bdnf* gene expression was silenced using CRISPR interference system (dCas9-KRAB) targeting *Bdnf* promoters I and IV. The neurons were treated at 8 DIV with 25 mM KCl for 6 h. Total *Bdnf* mRNA levels were measured by RT-qPCR. **(B)** BDNF protein levels in adult murine cerebral cortex and in BDNF-silenced and KCl-treated cultured cortical neurons along with recombinant proBDNF and mature BDNF were measured by Western blot. Note that the endogenous proBDNF in rat neurons has slower mobility compared to the bacterially expressed recombinant human proBDNF probably due to glycosylation. Red asterisk marks an unspecific signal. **(C)** The efficiency of the anti-BDNF 3C11 antibody to detect proBDNF and mature BDNF was also tested in HEK293 cells by overexpressing BDNF-V5 and performing Western blot with anti-V5 and anti-BDNF antibody. **(D,F,G,H)** The epitope of the anti-BDNF 3C11 antibody (Icosagen) was determined using mimotope variation analysis (MVA). x-axis indicates the amino acid position of mouse BDNF **(D)**, NGF **(F)**, NT-3, **(G)**, or NT-4 **(H)** protein (Uniprot IDs P21237, P01139, P20181, Q80VU4, respectively). Pre, pro and mature neurotrophin domains are shown below the graph. Alignment load for each amino acid position was calculated as the sum of normalized counts of aligning 12-mer peptides (with maximum 6 mismatches) obtained from MVA or from direct sequencing of the input phage library. y-axis depicts anti-BDNF antibody alignment load fold enrichment compared to the alignment load of the input library. Specific signal of antibody binding can be seen for amino acids _147_SEWVTAADK_155_, residing in the N-terminal region of mature BDNF. **(E)** Depiction of the identified epitope (Ser147-Lys155) on the 3D structure model of the mature BDNF protein (residues Ser132-Arg249). The epitope (in orange) of the 3C11 anti-BDNF antibody (combined cartoon and surface representation, gray). The protein model was obtained from the AlphaFold Protein Structure Database (AF-P21237-F1-model-v4) and visualized using PyMOL.

For Western blot analysis, we chose the 3C11 monoclonal anti-BDNF antibody (Icosagen) that has been previously validated using protein lysates of *Bdnf* knock-out animals (Andreska et al., 2020; Wosnitzka et al., 2020). We have previously shown that the 3C11 antibody can detect both proBDNF and mature BDNF in rat cultured neurons after prolonged depolarization (Esvald et al., 2022). To confirm the specificity of the signal seen in Western blot, we silenced the expression of *Bdnf* using CRISPR interference system targeted to *Bdnf* promoters I and IV in cultured cortical neurons (Figures 8A,B). CRISPR interference led to decreased levels of *Bdnf* mRNA and also proBDNF and mature BDNF protein as expected. We also show that this antibody can detect both mature and proBDNF with similar efficiency (Figure 8C). We conclude that the 3C11 BDNF antibody can recognize endogenous levels of both proBDNF and mature BDNF.

To further characterize the antibody, we performed antibody epitope mapping using mimotope variation analysis (MVA) (Sadam et al., 2018, 2021), a next generation phage display method that has been shown to be suitable for mapping the epitopes of purified antibodies (Kasak et al., 2017). Our MVA results indicate that the epitope for the 3C11 anti-BDNF antibody consists of amino acids _147_SEWVTAADK_155_ in the mature region of the BDNF protein (Figures 8D,E). Furthermore, this region in BDNF protein is 100% conserved between mouse, rat, human and other mammals. In addition, we detected no major cross-reactivity of the anti-BDNF antibody with other neurotrophic factors using MVA (Figures 8F–H).

**Figure 9:**
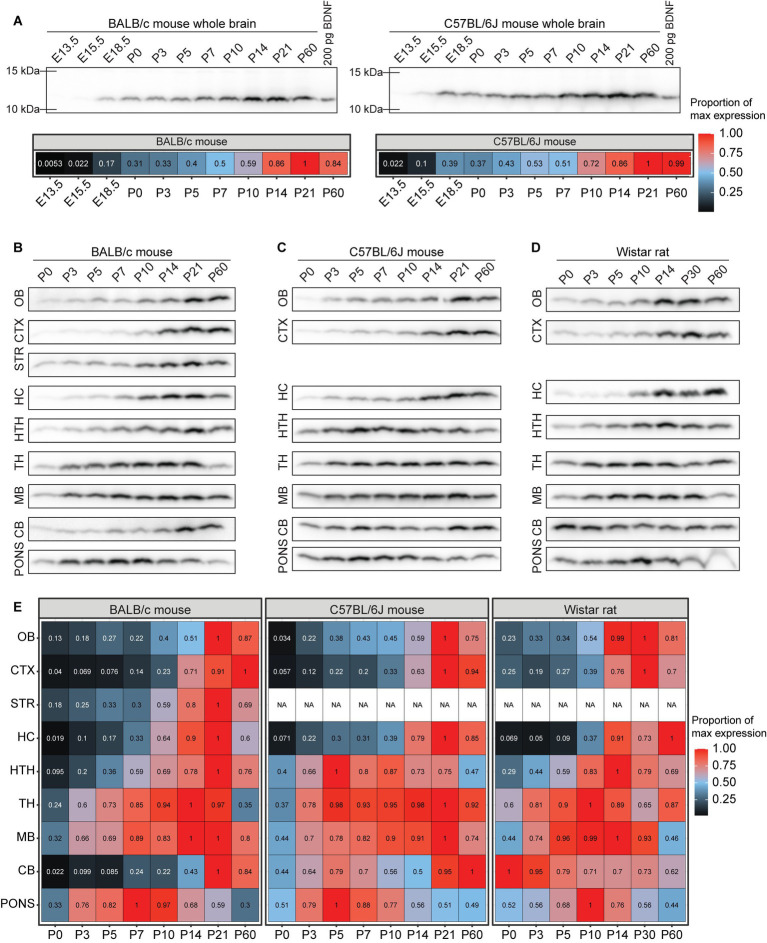
The dynamics of BDNF protein expression in BALB/c mouse, C57BL/6 J mouse and Wistar rat brain regions during development. BDNF protein levels measured by Western blot in BALB/c and C57BL/6 J mouse, and Wistar rat different brain regions throughout development. Fifty micro gram total protein lysate was loaded per lane. **(A)** BDNF protein levels in the whole brain of BALB/c and C57BL/6 J mice (upper panel) and densitometric quantification (lower panel). The maximal BDNF expression level in the respective mouse line in the respective brain region was set as 1. BDNF protein levels in different brain regions of **(B)** BALB/c mouse, **(C)** C57BL/6 J mouse, and **(D)** Wistar rat during development. **(E)** Densitometric quantification of BDNF protein expression levels. The maximal BDNF expression level in the respective animal model brain region was set as 1. E – embryonic day, P – postnatal day, OB – olfactory bulb, CTX – cerebral cortex, STR – striatum, HC – hippocampus, HTH – hypothalamus, TH – thalamus, MB – midbrain, CB – cerebellum, NA – not analyzed.

Our results show that in the whole brain of BALB/c and C57BL/6 J mice BDNF protein levels gradually increase, peak during the third postnatal week, and remain high in adulthood (Figure 9A). In most BALB/c brain regions BDNF protein levels peak at the second or third postnatal week, while the pons shows an earlier peak at P7 (Figures 9B,E). In C57BL/6 J mouse, BDNF protein levels peak at third postnatal week in most regions, although in the hypothalamus, thalamus, and pons the peak is already seen at P5 (Figures 9C,E). In Wistar rat, the levels of BDNF peak at P10-P30 in most brain regions, although the highest BDNF protein expression in the cerebellum was seen at P0 (Figures 9D,E).

**Figure 10:**
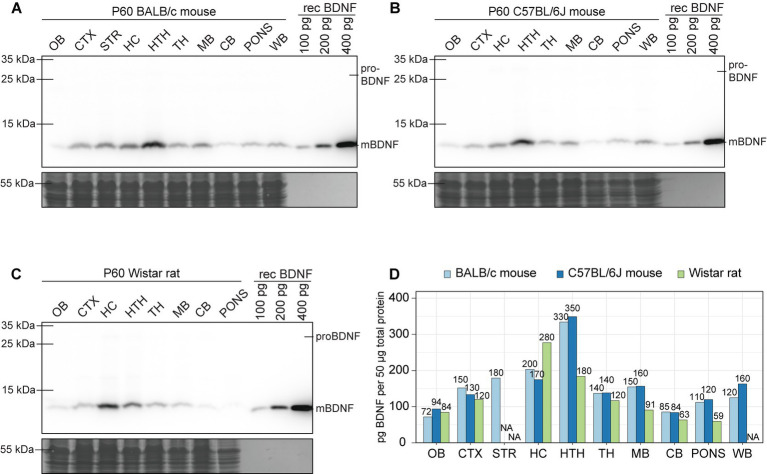
The expression of BDNF protein in adult BALB/c and C57BL/6 J mice, and Wistar rat brain regions. Shown are BDNF protein levels measured by Western blot in BALB/c **(A)** and C57BL/6 J **(B)** mouse, and Wistar rat **(C)** different brain regions at postnatal day 60 (P60). Fifty micro gram total protein lysate was loaded per lane. **(D)** Densitometric quantification of BDNF protein and calculated amounts using calibration curve based on recombinant BDNF protein levels. P – postnatal day, OB – olfactory bulb, CTX – cerebral cortex, STR – striatum, HC – hippocampus, HTH – hypothalamus, TH – thalamus, MB – midbrain, CB – cerebellum, WB – whole brain, NA – not analyzed.

Next, we analyzed BDNF protein levels among different brain regions of adult murines. In both BALB/c and C57BL/6 J mice the highest levels of BDNF protein are found in the hypothalamus (Figures 10A,B,D) and in Wistar rat the highest levels are in the hippocampus (Figures 10C,D). In all studied animal models, the lowest levels of BDNF protein are in olfactory bulb and cerebellum (Figure 10). Notably, only mature BDNF protein was present in the brain, and virtually no proBDNF protein could be detected. The levels of proBDNF seem to be at least an order of magnitude lower than levels of mature BDNF in adult murine brain (Figures 10A–C). Collectively, our results show complex spatiotemporal regulation of the expression of BDNF protein in murine brain.

### BDNF protein levels in murine non-neural tissues during development

**Figure 11:**
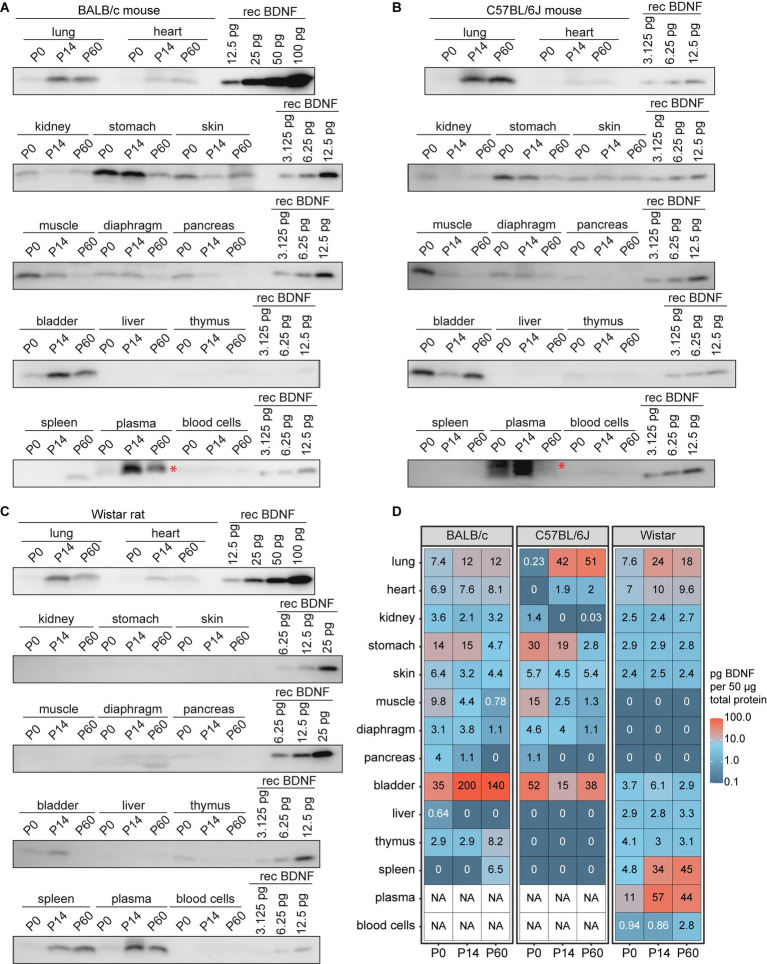
BDNF protein levels in non-neural tissues of BALB/c and C57BL/6 J mice and Wistar rat during development. BDNF protein levels measured by Western blot in BALB/c **(A)** and C57BL/6 J **(B)** mouse, and Wistar rat **(C)** non-neural tissues throughout development. Fifty micro gram total protein lysate was loaded per lane. **(D)** Quantification of BDNF protein levels based on recombinant BDNF (rec BDNF) signals that were used to calculate calibration curve for each blot. Numbers in the heatmap show BDNF protein quantity in pg. per 50 μg of total protein. Red asterisk marks an unspecific signal in mice plasma and blood cells, possibly masking the BDNF protein signal **(A,B)** and therefore no quantification of BDNF protein was performed [NA in white boxes in panel **(D)**]. NA – not available, P – postnatal day.

As the RNA sequencing analysis showed *BDNF* expression in numerous non-neural tissues and various roles for BDNF in non-neural tissues have been shown (see Introduction for references), we finally analyzed BDNF protein levels in the non-neural tissues of BALB/c and C57BL/6 J mice and Wistar rat. First, our results show that in BALB/c and C57BL/6 J mice and Wistar rats, BDNF protein levels in non-neural tissues are generally lower than those detected in most brain regions, e.g., being barely detectable in the liver and thymus, regardless of the developmental stage (Figures 10, 11D). C57BL/6 J mouse and Wistar rat, and at lower levels also BALB/c mouse, all show expression of BDNF protein in the lung during postnatal development, with virtually no levels at P0 and relatively high levels at P14 and P60 (Figure 11). Second, in both mouse strains, the bladder and stomach were among the non-neural tissues with the highest BDNF protein levels, which tend to decrease during postnatal development (Figures 11A,B,D), whereas Wistar rat show minimal BDNF protein in these tissues (Figures 11C,D). The opposite can be seen for the spleen, which shows very low BDNF protein levels in mice (Figures 11A,B,D), whereas BDNF protein can be readily detected in Wistar rat (Figures 11C,D). In the skeletal muscle BDNF protein was seen at P0 but not at P14 and P60 in both mouse strains. In Wistar rat skeletal muscle BDNF protein was not detected during postnatal development. As it has been reported that there is no BDNF protein in mouse blood (Radka et al., 1996), and the strong Western blot signals in mouse plasma is showing a slightly higher apparent molecular weight (denoted with red asterisks), we conclude that these signals are unspecific. However, we detected BDNF protein with correct apparent molecular weight in Wistar rat plasma (Figure 11), in agreement with the previously published results (Radka et al., 1996; Chacón-Fernández et al., 2016). Altogether, our results show BDNF protein expression in many mouse and rat non-neural tissues, although the protein levels in different murines are not as consistent as in the central nervous system.

## Discussion

In the current study we used RNA sequencing data from over 3,600 samples across 18 published datasets, and over 17,000 samples from GTEx, and approximately 180 samples from the BrainSpan database to generate a comprehensive understanding of *BDNF*, *TRKB*, and *P75NTR* mRNA expression in different mammalian tissues, complementing the previous studies (Hofer et al., 1990; Timmusk et al., 1993a,b, 1994a,b; Conner et al., 1997; Croll et al., 1998; Das et al., 2001; Stoilov et al., 2002; Silhol et al., 2005; Aid et al., 2007; Pruunsild et al., 2007; Kumanogoh et al., 2008; Luberg et al., 2010; West et al., 2014; Pattwell et al., 2022). Furthermore, using a highly specific and sensitive antibody capable of detecting low picogram levels of BDNF, we complemented the bioinformatically obtained data by thoroughly characterizing BDNF protein levels in both the nervous system and non-neural tissues throughout mouse and rat development.

The regulation of *Bdnf* gene expression has primarily been studied in the cerebral cortex and hippocampus, where BDNF is highly expressed (West et al., 2014). We (Esvald et al., 2022) and others (Flavell et al., 2008; McDowell et al., 2010; Lyons et al., 2012) have shown brain region-specific differences in *Bdnf* gene regulation between the cerebral cortex and hippocampus. For instance, *Bdnf* expression in the cortex is regulated by CaRF (McDowell et al., 2010), and potentially by FOXP1, SATB family members, and BCL11A (Esvald et al., 2022). In contrast, in the hippocampus, *Bdnf* expression is regulated by MEF family members (Flavell et al., 2008; Lyons et al., 2012) and possibly by PROX1 (Esvald et al., 2022). Our results here demonstrate high *Bdnf* mRNA and protein expression also in other brain regions, e.g., in the hypothalamus, thalamus, midbrain, and pons, in addition to the cerebral cortex and hippocampus. It is plausible that there are brain region and/or cell type-specific regulatory mechanisms involved. Unfortunately, the mechanisms of *Bdnf* gene regulation in these brain regions have not been studied thoroughly. To the best of our knowledge, previously only the zinc finger transcription factor retinoic acid-induced 1 (RAI1) has been shown to regulate *Bdnf* expression in the hypothalamus (Burns et al., 2010; Huang et al., 2016; Javed et al., 2021; Esvald et al., 2022), and the nuclear receptor subfamily 4 group A member 2 (NR4A2, also known as NURR1) in midbrain neurons (Volpicelli et al., 2007), cerebellar granule neurons (Barneda-Zahonero et al., 2012) and hippocampal neurons (Català-Solsona et al., 2023), but not in cortical neurons (Abdollahi and Fahnestock, 2022). We hypothesize that in addition to widely expressed transcription factors, novel tissue- and cell-type specific regulators control the expression of *Bdnf* gene in these brain regions.

In addition to the brain, important roles for BDNF have recently been described in many non-neural tissues (see the Introduction section for more information and references). Adding to the previously known non-neural tissues where *Bdnf* is expressed, e.g., heart, muscle, lung, kidney (Timmusk et al., 1993a; Aid et al., 2007), we extend this list by reporting *Bdnf* expression in murine esophagus, forestomach, skin, and adipose tissues, and in human circulatory system, prostate, bladder, pituitary gland, and fibroblasts. Considering the high levels of *TRKB-T1* mRNA in circulatory and integumentary system, our results warrant further studies of BDNF functions in these tissues. In addition, we show that in murines, the highest BDNF protein levels are found in lung, heart, stomach, and bladder. Notably, according to single-cell data, *BDNF* expression is limited to specific cell types in many tissues, such as fibroblasts in the adult mouse heart. Our analysis also reveals that *BDNF* is expressed by various cell types in the vascular system. Altogether, our results imply that BDNF can be produced only by certain specialized cell populations and still possibly play a crucial role in the proper development and/or functioning of an organ.

Already more than 20 years ago it was shown that *Bdnf* homozygous knock-out mice die within weeks after birth due to cardiac failure (Donovan et al., 2000). Since then, BDNF has been shown to play an important role in heart function in different mouse models (Li et al., 2022; Yang et al., 2022). However, our analysis reveals contrasting developmental expression dynamics of *BDNF* in the heart across different organisms, with an increase in *Bdnf* mRNA levels in murines and a decrease in humans. Considering this non-conserved expression pattern, further studies are needed to clarify the precise function and expression of *BDNF* in the developing and adult human heart. It would also be of interest to study the functional role of BDNF in the bladder, where its biological functions have not yet been described. In fact, currently no gene regulatory mechanism for *BDNF* expression has been identified in non-neural tissues. Therefore, it would be also interesting to explore the regulation of *BDNF* expression in the specific cell populations that express *BDNF* in the non-neural tissues.

*BDNF* gene contains several different promoters that direct the expression of *BDNF* transcripts with different 5’ UTRs (Aid et al., 2007; Pruunsild et al., 2007). The existence of different promoters has been shown to provide tissue-specific expression of *Bdnf* (Timmusk et al., 1993b). Although the expression of different *Bdnf* transcripts have been analyzed in different tissues in both murines (Aid et al., 2007) and humans (Pruunsild et al., 2007), the published semiquantitative RT-PCR results do not allow direct comparison of the relative levels of different *Bdnf* transcripts. Our comprehensive analysis provides, for the first time, the proportions of different transcripts and how they contribute to total *BDNF* mRNA levels throughout development in different mammalian tissues. We found that different *BDNF* transcripts contribute to the total *BDNF* mRNA pool in different brain regions. For example, the most prevalent transcripts in the hypothalamus contain exon I, in the cerebellum exon II, in the cerebral cortex exon IV, and in the amygdala and thalamus one third of *BDNF* transcripts contain exon I. This is in agreement with previous results demonstrating that loss of BDNF synthesis from specific *Bdnf* transcripts differentially reduces BDNF protein levels across brain regions (Maynard et al., 2016). Our results imply that different mechanisms regulate specific *BDNF* transcripts in a brain region- and tissue-dependent manner, potentially providing the foundation for the distinct biological functions of different *BDNF* transcripts (see Introduction).

In agreement with previously published findings (Aid et al., 2007; Pruunsild et al., 2007), we also demonstrate that the expression of *BDNF* exon I, II, and III transcripts is mainly brain-specific, whereas exon IV and VI transcripts are expressed in both neural and non-neural tissues. Our results reveal distinct tissue-specific usage of alternative *BDNF* transcripts, emphasizing the specialized regulatory mechanisms of *BDNF* expression across various tissues. This possibly arises from the specific cell types expressing distinct *BDNF* transcripts. For example, it has been shown that neurons express all major *BDNF* transcripts, whereas astrocytes express only exon IV and VI-containing transcripts (Koppel et al., 2018). The limited expression of *BDNF* exons I-III possibly results from the combination of active repression in non-neuronal cells by neuron-restrictive silencer factor (NRSF) that binds *BDNF* promoter II (Schoenherr and Anderson, 1995; Timmusk et al., 1999; Zuccato et al., 2003), and a neuron-specific enhancer increasing the expression of the aforementioned transcripts (Tuvikene et al., 2021). Further studies are needed to elucidate the temporal, cell- and transcript-specific pattern of *BDNF* expression in different brain regions as well as non-neural tissues in various physiological and pathophysiological conditions.

It has been proposed that the evolution of gene regulatory regions drives the differences in gene expression between species and underlies the evolution of complex phenotypes (Wilson and Odom, 2009; Romero et al., 2012). During evolution, the human *BDNF* gene has acquired additional exons and potential translation initiation sites compared to the murine *Bdnf* gene (Pruunsild et al., 2007). Furthermore, *BDNF* proximal promoters contain evolutionarily non-conserved *cis*-elements that result in different stimulus-dependent regulation, such as the CRE element in the human *BDNF* promoter IX which is absent from the murine promoter but is present in primates (Esvald et al., 2020). In contrast, the mature BDNF protein amino acid sequence is highly conserved among mammals, indicating the functional importance of BDNF protein (Lucaci et al., 2022). Here, we show that total *BDNF* mRNA levels are similar, while the usage of different *BDNF* promoter regions varies between mammals, suggesting evolutionary changes in proximal promoter and/or enhancer regions. Although there seem to be tissue-specific differences in the usage of 5′ exons, the usage of alternative polyadenylation signals and the resulting 3’ UTRs are conserved throughout mammalian evolution. Notably, as total *BDNF* mRNA levels in the brain are roughly similar in mammals (except for opossum), the upregulation of certain *BDNF* transcripts seems to result in downregulation of other *BDNF* transcripts, implying remarkable feedback loops for maintaining stable total *BDNF* expression levels.

In this study, we provide a comprehensive description of BDNF protein levels in murine brain regions and non-neural tissues. Our results show that BDNF protein levels peak at P21 and at P10-14 in most mouse and rat brain regions, respectively. In adult murines, the highest levels of BDNF protein are found in the hippocampus and hypothalamus, which is in agreement with the well-established role of BDNF in memory and learning processes in the hippocampus (Park and Poo, 2013), and metabolic control in the hypothalamus (Unger et al., 2007; An et al., 2015). Interestingly, our results also indicate that *Bdnf* mRNA levels do not always reflect BDNF protein levels. For example, while the total *Bdnf* mRNA levels in mouse hypothalamus and cortical regions are similar, the BDNF protein levels in the hypothalamus are much higher. This discrepancy could be explained by high proportion of exon I-containing *Bdnf* transcripts, which exhibit higher translation efficiency (Koppel et al., 2015), and low proportion of *Bdnf* transcripts with long 3’ UTR, which are less associated with polysomes than transcripts with short 3’ UTR (Timmusk et al., 1994b) in the hypothalamus. However, we cannot exclude the possibility of BDNF protein transport into the hypothalamus from other brain regions projecting to it (Saper, 2000). Similarly, we observe relatively strong *Bdnf* mRNA expression in the cerebellum, although the BDNF protein levels in this brain region are among the lowest in the brain. This difference might be due to the very low proportion of the well-translated *Bdnf* exon I transcripts, high proportion of the poorly translated exon II-containing transcripts (Lekk et al., 2023), and a relatively high proportion of transcripts with long 3’ UTR, possibly further impairing BDNF translation. Furthermore, total *Bdnf* mRNA levels in the lung and brain are comparable, but BDNF protein levels are significantly lower in the lung. Based on our data, we hypothesize that BDNF protein levels are a subject to complex regulation involving 5′ and 3’ UTRs as well as interregional transportation.

Collectively, our results provide and extensive description of *BDNF*, *TRKB*, and *P75NTR* mRNA expression and BDNF protein levels. This comprehensive analysis not only confirms but also broadens the existing knowledge, serving as a valuable input for future research avenues.

## Data availability statement

The original contributions presented in the study are included in the Supplementary material. The datasets presented in this study can be found in online repositories. The names of the repositories and accession number(s) can be found in the Supplementary material. Further inquiries can be directed to the corresponding author.

## Ethics statement

The animal study was reviewed and approved by Ministry of Agriculture of Estonia (Permit Number: 45).

## Author contributions

E-EE designed research, performed Western blot, wrote the first draft, and edited the manuscript. JT designed research, performed bioinformatical analysis, analyzed MVA experiment, and wrote and edited the manuscript. CK designed research, performed bioinformatical analysis and Western blot and edited the manuscript. AA, FC-C, LT, and IK performed Western blot and edited the manuscript. ASi, ASh, and LT provided tissue lysates and edited the manuscript. AP designed and analyzed MVA experiment. KP designed the MVA experiment, provided funding, and reviewed the manuscript. TT conceived the idea, supervised the study, provided funding, and reviewed the manuscript. All authors contributed to the article and approved the submitted version.

## Funding

This study was supported by the Estonian Research Council (grants PRG805 to TT, PRG573 and PRG1953 to KP), European Union through the European Regional Development Fund (project no. 2014–2020.4.01.15–0012 to TT), H2020-MSCA-RISE-2016 (grant EU734791 to KP and TT) and European Commission and Estonian Research Council (ERA-NET NEURON Cofund2 programme grant GDNF UpReg to TT and KP). JT was partially funded by the Estonian Ministry of Education and Research grant 2014–2020.4.01.21–0315.

## Conflict of interest

E-EE, JT, AP, KP, and TT were employed by Protobios LLC. JT was employed by dxlabs LLC.

The remaining authors declare that the research was conducted in the absence of any commercial or financial relationships that could be construed as a potential conflict of interest.

## Publisher’s note

All claims expressed in this article are solely those of the authors and do not necessarily represent those of their affiliated organizations, or those of the publisher, the editors and the reviewers. Any product that may be evaluated in this article, or claim that may be made by its manufacturer, is not guaranteed or endorsed by the publisher.

## Footnotes

^1^ https://www.proteinatlas.org/

^2^ https://gtexportal.org

^3^ http://brainspan.org
