# Supplementary material for: Revisiting the expression of BDNF and its receptors in mammalian development
Source: Front Mol Neurosci. 2023 Jun 22;16:1182499. doi: 10.3389/fnmol.2023.1182499 (PMC10325033; doi:10.3389/fnmol.2023.1182499)
Supplement: Supplementary file 1 [file Presentation_1.pdf]

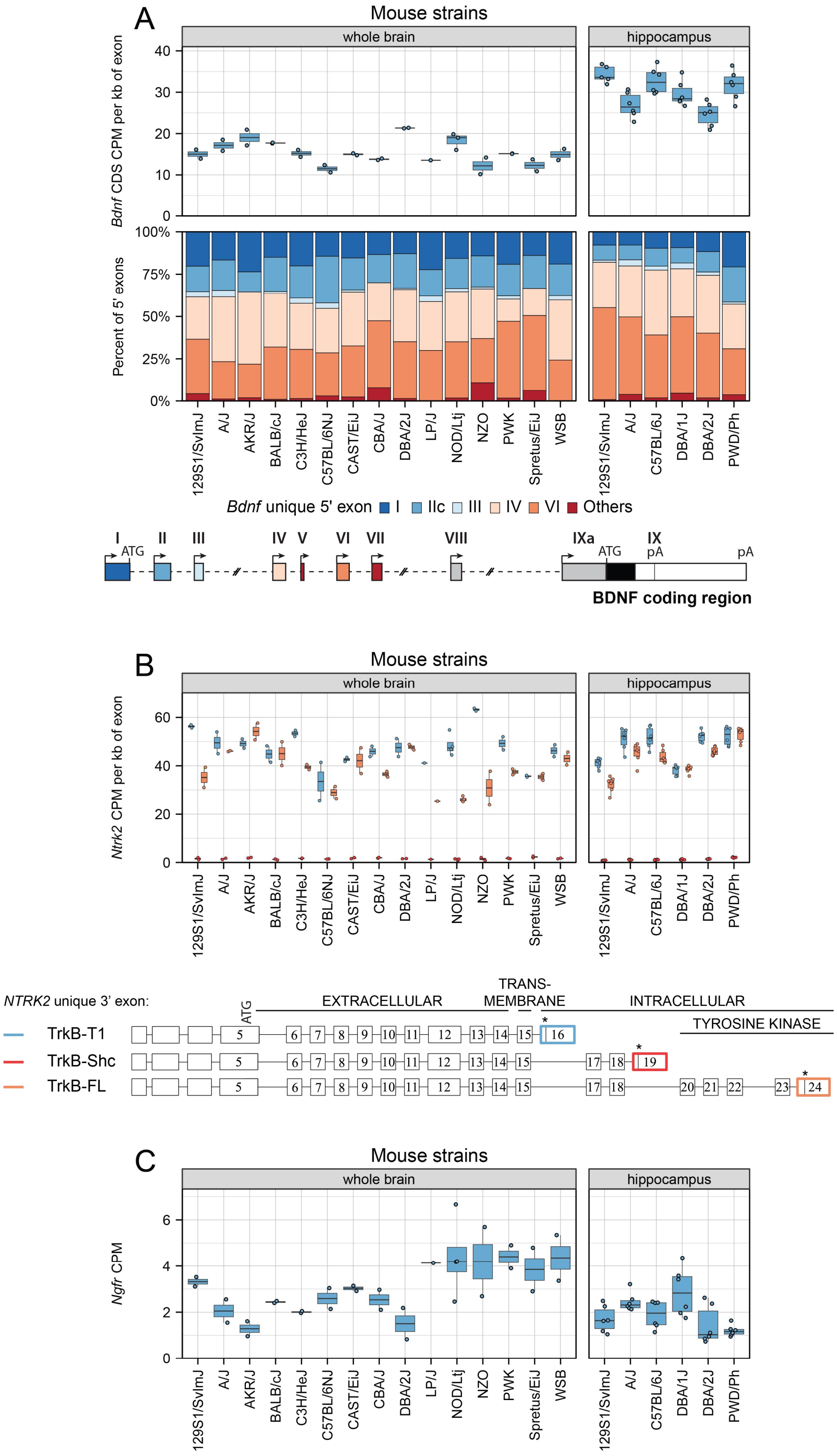

Supplementary figure S1. Comparison of *Bdnf*, *TrkB*, and *p75NTR* levels in different adult mouse strains. Meta-analysis of *Bdnf*, *Ntrk2* (encoding TrkB) and *Ngfr* (encoding p75NTR) expression levels in different adult (postnatal day 56-64) mouse strain whole brain and hippocampus. **(A)** Total *Bdnf* expression measured by levels of *Bdnf* coding sequence (CDS, upper panel) and distribution of levels of *Bdnf* 5' exons (lower panel) are shown as depicted on the schematics of murine gene structure. The exons indicated with grey color were not included in the analysis of 5' exons. **(B)** The mRNA levels of different *TrkB* isoforms based on the levels of unique 3' exons of *Ntrk2* gene (mouse counterpart of human exon 16, 19, 24). The 3' exons specific for *TrkB* isoforms *TrkB-T1*, *TrkB-Shc*, and *TrkB-FL* are shown on the scheme (adapted from Luberg et al. (2010)). Asterisks on the schematics mark stop-codons. **(C)** Total *Ngfr* mRNA expression levels. All individual animals are shown as dots, the hinges show 25% and 75% quartiles, where the horizontal line shows the median value, the upper whisker extends from the hinge to the largest value no more than 1.5 of the inter-quartile range, and the lower whisker extends from the hinge to the smallest value at most 1.5 of the inter-quartile range of the hinge. All used datasets and underlying data are shown in Supplementary table 4. CDS – coding sequence, CPM – counts per million.

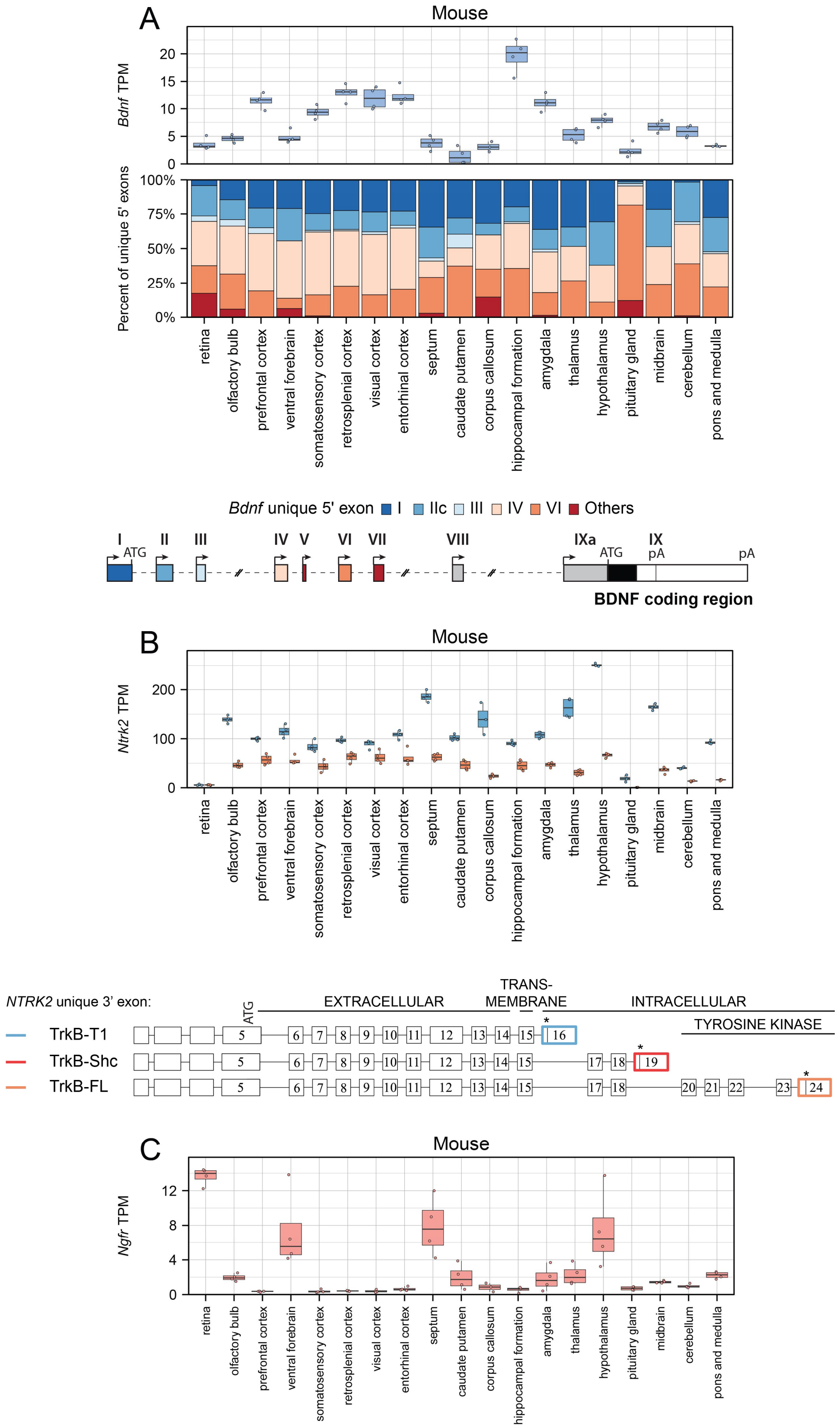

Supplementary figure S2. Comparison of *Bdnf*, *TrkB* and *p75NTR* levels in different adult mouse brain regions. Data visualization of *Bdnf*, *Ntrk2* (encoding TrkB) and *Ngfr* (encoding p75NTR) expression levels in adult (postnatal day 60) mouse brain regions (ordered from rostral to caudal) from The Human Protein Atlas. **(A)** Total *Bdnf* expression levels (upper panel) and distribution of unique *Bdnf* 5’ transcripts (lower panel) are shown as depicted on the schematics of murine gene structure. The exons indicated with grey color were not included in the analysis of 5’ exons. **(B)** The mRNA levels of different *TrkB* isoforms based on the levels of unique 3’ exons of *Ntrk2* gene (mouse counterpart of human exon 16, 19, 24). The 3’ exons specific for *TrkB* isoforms *TrkB-T1*, *TrkB-Shc*, and *TrkB-FL* are shown on the schematics (adapted from Luberg et al. (2010)). Asterisks on the scheme mark stop-codons. **(C)** Total *Ngfr* mRNA expression levels. All individual animals are shown as dots, the hinges show 25% and 75% quartiles, where the horizontal line shows the median value, the upper whisker extends from the hinge to the largest value no more than 1.5 of the inter-quartile range, and the lower whisker extends from the hinge to the smallest value at most 1.5 of the inter-quartile range of the hinge. All used datasets and underlying data are shown in Supplementary table 5. TPM – transcripts per million.

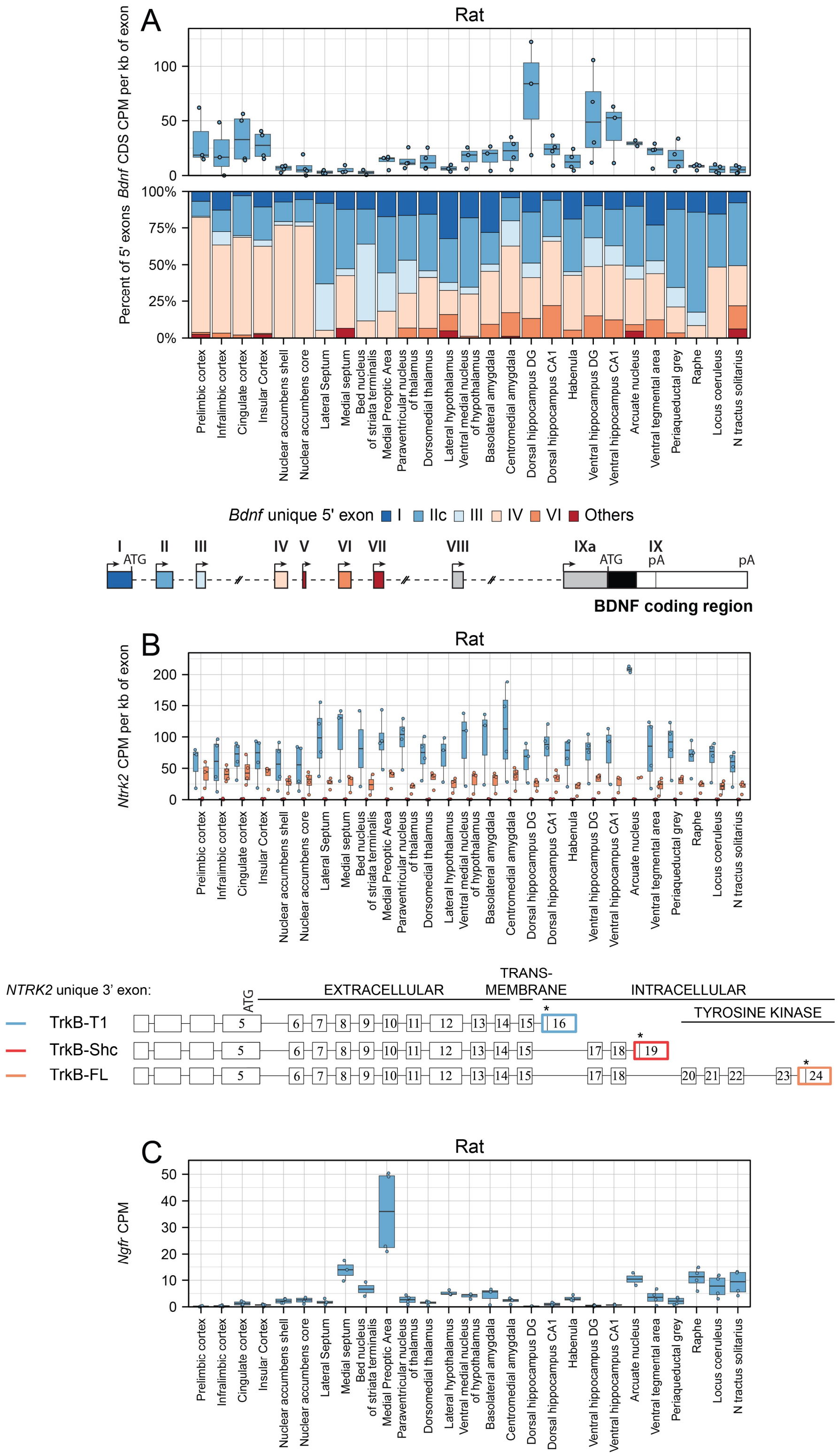

Supplementary figure S3. Comparison of *Bdnf*, *TrkB* and *p75NTR* levels in different adult rat brain regions. Data visualization of *Bdnf*, *Ntrk2* (encoding TrkB) and *Ngfr* (encoding p75NTR) expression levels in adult (postnatal day 60) rat brain regions (ordered from rostral to caudal) from (Rayan et al., 2022). **(A)** Total *Bdnf* expression levels (upper panel) and distribution of unique *Bdnf* 5' transcripts (lower panel) are shown as depicted on the schematics of murine gene structure. The exons indicated with grey color were not included in the analysis of 5' exons. **(B)** The mRNA levels of different *TrkB* isoforms based on the levels of unique 3' exons of *Ntrk2* gene (mouse counterpart of human exon 16, 19, 24). The 3' exons specific for *TrkB* isoforms *TrkB-T1*, *TrkB-Shc*, and *TrkB-FL* are shown on the schematics (adapted from Luberg et al. (2010)). Asterisks on the scheme mark stop-codons. **(C)** Total *Ngfr* mRNA expression levels. All individual animals are shown as dots, the hinges show 25% and 75% quartiles, where the horizontal line shows the median value, the upper whisker extends from the hinge to the largest value no more than 1.5 of the inter-quartile range, and the lower whisker extends from the hinge to the smallest value at most 1.5 of the inter-quartile range of the hinge. All used datasets and underlying data are shown in Supplementary table 6. CPM – counts per million.

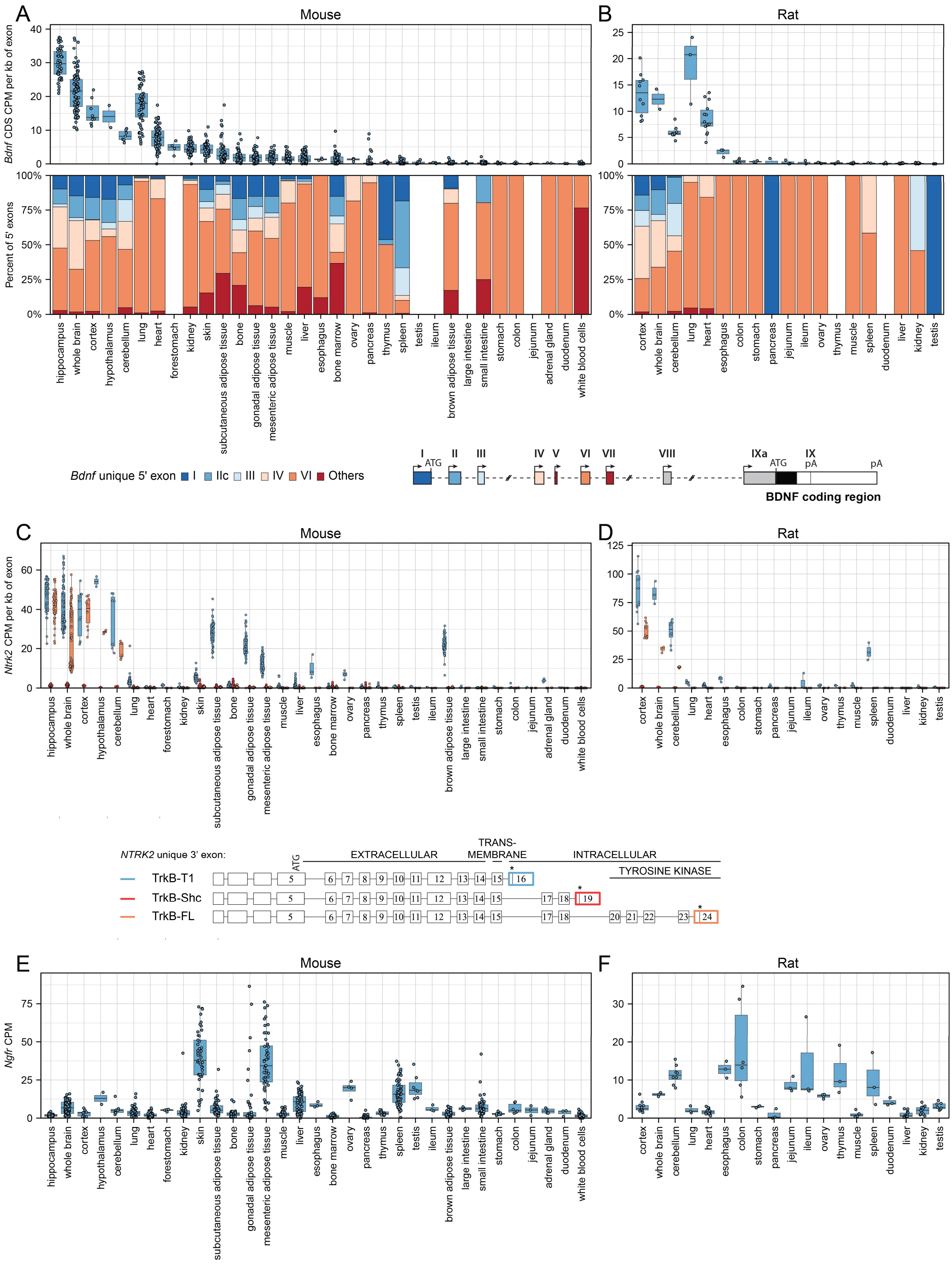

Supplementary figure S4. The expression levels of *Bdnf* and its receptors *TrkB* and *p75NTR* in adult mouse and rat brain regions and non-neural tissues. Meta-analysis of *Bdnf*, *Ntrk2* (encoding TrkB) and *Ngfr* (encoding p75NTR) expression levels in adult (postnatal day 42+) mouse **(A, C, E)** and rat **(B, D, F)** brain and non-neural tissues (ordered from highest to lowest total *Bdnf* mRNA levels). **(A, B)** Total *Bdnf* levels measured

by levels of *Bdnf* coding sequence (CDS, upper panel) and distribution of levels of *Bdnf* 5' exons (lower panel) are shown as depicted on the schematics of murine gene structure. The exons indicated with grey color were not included in the analysis of 5' exons. White box in the proportion of *Bdnf* 5' exons indicates that the expression of *Bdnf* was too low to calculate the proportion of 5' exons. **(C, D)** The mRNA levels of different *TrkB* isoforms based on the levels of unique 3' exons of *Ntrk2* gene (mouse or rat counterpart of human exon 16, 19, 24). The 3' exons specific for *TrkB* isoforms *TrkB-T1*, *TrkB-Shc*, and *TrkB-FL* are shown on the scheme (adapted from Luberg et al. (2010)). Asterisks on the schematics mark stop-codons. **(E, F)** Total *Ngfr* mRNA expression levels. All individual animals are shown as dots, the hinges show 25% and 75% quartiles, where the horizontal line shows the median value, the upper whisker extends from the hinge to the largest value no more than 1.5 of the inter-quartile range, and the lower whisker extends from the hinge to the smallest value at most 1.5 of the inter-quartile range of the hinge. All used datasets and underlying data are shown in Supplementary table 8. CDS – coding sequence, CPM – counts per million.

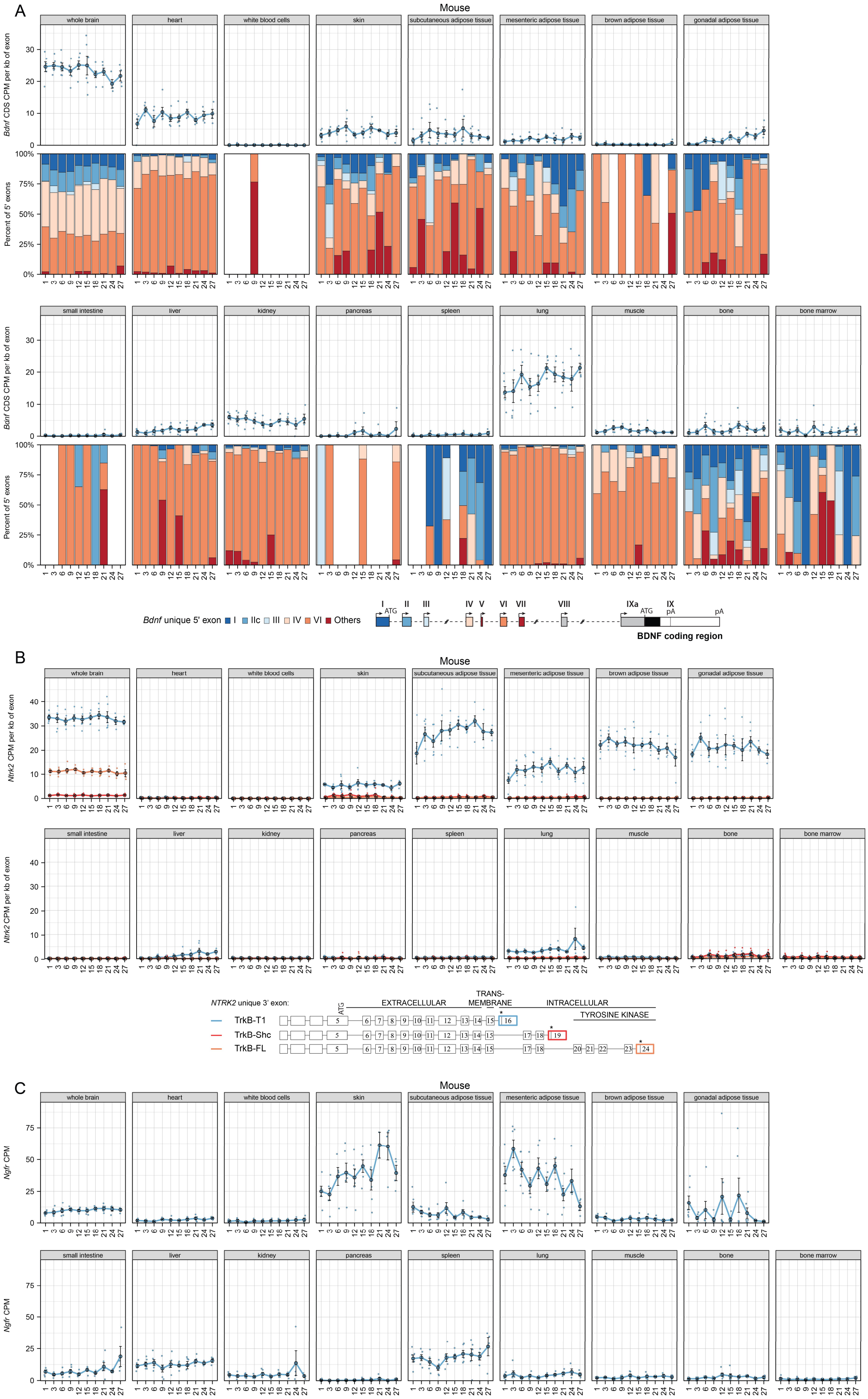

Supplementary figure S5. The expression levels of *Bdnf* and its receptors *TrkB* and *p75NTR* during mouse lifespan. Data visualization of *Bdnf*, *Ntrk2* (encoding TrkB) and *Ngfr* (encoding p75NTR) expression levels in 1 to 27 months old mouse brain and non-neural tissues from Tabula Muris Consortium. **(A)** Total *Bdnf* expression levels (upper panel) and distribution of unique *Bdnf* 5' transcripts (lower panel) are shown as depicted

on the schematics of murine gene structure. The exons indicated with grey color were not included in the analysis of 5' exons. **(B)** The mRNA levels of different *TrkB* isoforms based on the levels of unique 3' exons of *Ntrk2* gene (mouse counterpart of human exon 16, 19, 24). The 3' exons specific for *TrkB* isoforms *TrkB-T1*, *TrkB-Shc*, and *TrkB-FL* are shown on the schematics (adapted from Luberg et al. (2010)). Asterisks on the scheme mark stop-codons. **(C)** Total *Ngfr* mRNA expression levels. Data from individual animals are shown as small dots, circles indicate mean values and error bars represent standard error of the mean (SEM). All used datasets and underlying data are shown in Supplementary table 9. CDS – coding sequence, CPM – counts per million.

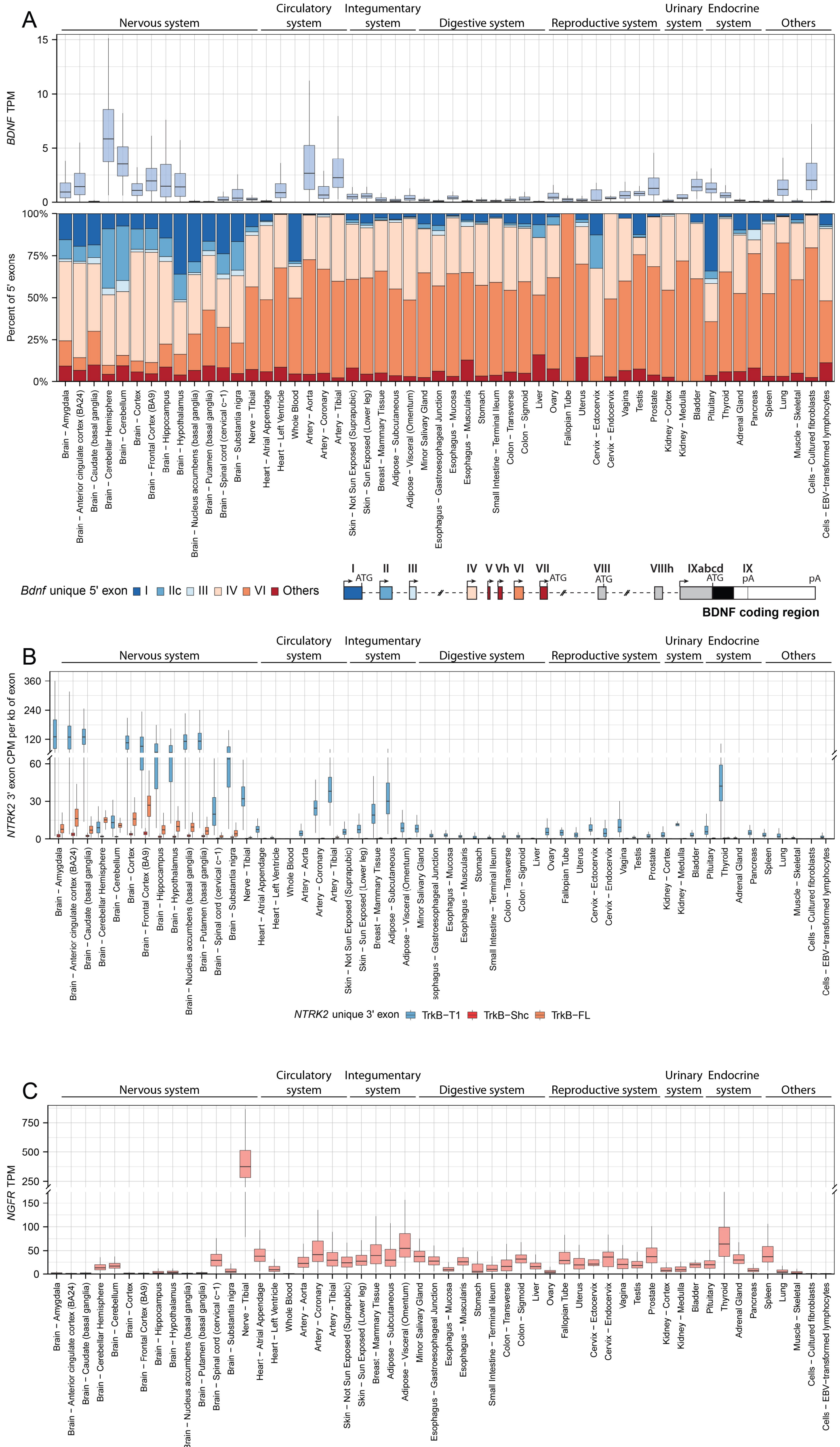

Supplementary figure S6. Comparison of *BDNF*, *TRKB* and *P75NTR* levels in different adult human brain regions and non-neural tissues. Visualization of *BDNF*, *NTRK2* (encoding TRKB) and *NGFR* (encoding P75NTR) expression levels in adult (20 to 80 years) human tissues from the Genotype-Tissue Expression (GTEx) project. **(A)** Total *BDNF* expression levels (upper panel) and distribution of unique *BDNF* 5' exons

(lower panel) are shown as depicted on the schematics of human gene structure. **(B)** The mRNA levels of different *TrkB* isoforms based on the levels of unique 3' exons of *NTRK2* gene (mouse counterpart of human exon 16, 19, 24). The 3' exons specific for *TRKB* isoforms *TRKB-T1*, *TrkB-SHC*, and *TRKB-FL* are shown on the schematics (adapted from Luberg et al. (2010)). Asterisks on the schematics mark stop-codons. **(C)** Total *NGFR* mRNA expression levels. The hinges show 25% and 75% quartiles, where the horizontal line shows the median value, the upper whisker extends from the hinge to the largest value no more than 1.5 of the inter-quartile range, and the lower whisker extends from the hinge to the smallest value at most 1.5 of the inter-quartile range of the hinge. All used datasets and underlying data are shown in Supplementary table 12. TPM – transcripts per million.

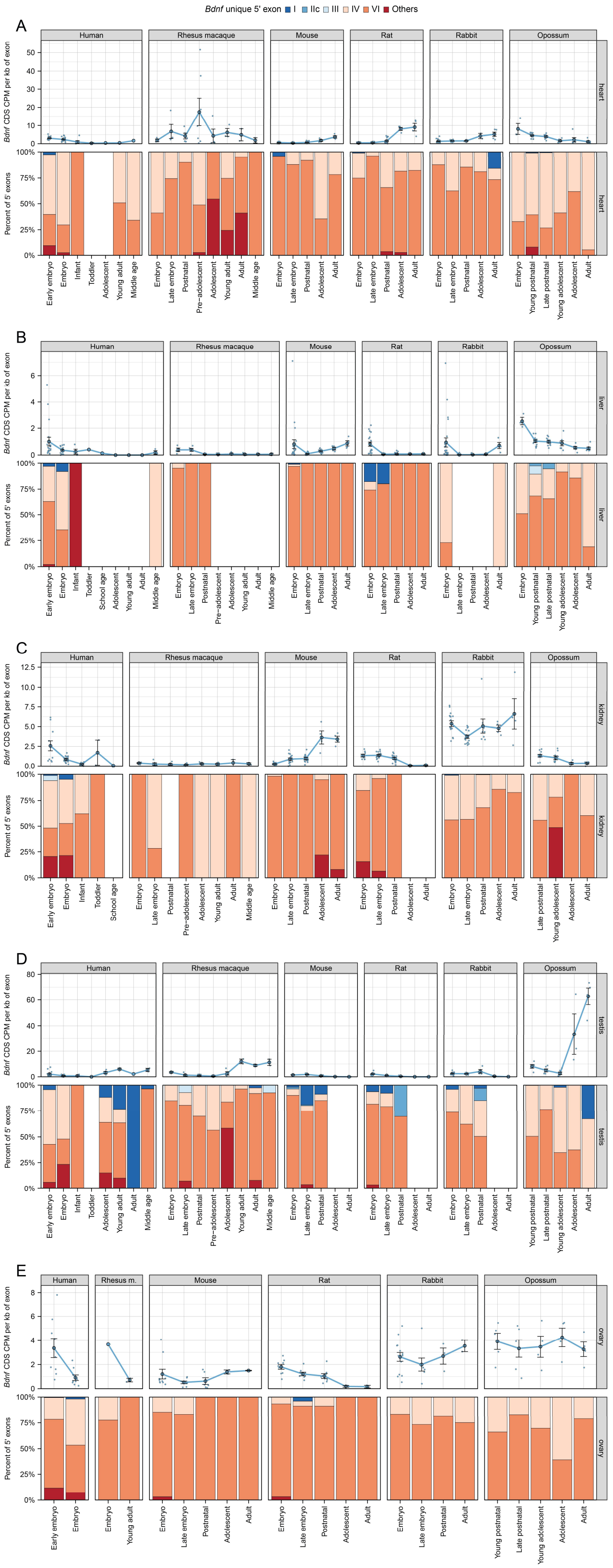

Supplementary figure S7. Comparison of *BDNF* mRNA levels in different mammals. Total *BDNF mRNA* levels measured by levels of *BDNF* coding sequence (CDS, upper panels) and distribution of levels of *BDNF* 5’ exons (lower panels) are shown in heart **(A)**, liver **(B)**, kidney **(C)**, testis **(D)**, and ovary **(E)**. The *BDNF* levels in humans are shown in early embryo (0-9 postcoital week (PCW), embryo (10-19 PCW), late embryo

(20-39 PCW), infant (younger than 12 months), toddler (1-4 years old), school age (7-8 years old), adolescent (10-17 years old), young adult (18-29.99 years old), adult (30-39.99 years old), and middle-aged (40-58 years old); in rhesus macaques in embryo (E93-E109), late embryo (E112-E130), postnatal (P0-P24), pre-adolescent (0.5-1 years old), adolescent (2-3 years old), young adult (8-11 years old), adult (14-15 years old), and middle-aged (20-26 years old) animals; in mouse and rats in embryo (E10.5-E14.5/E11-E15 mouse/rat), late embryo (E15.5-E18.5/E17-E20 mouse/rat), postnatal (P0-P14), adolescent (P22-56), and adult (P62+) animals; in rabbits in embryo (E12-E19.5), late embryo (E21-E27), postnatal (P10-P14), adolescent (P84), and adult (P186-P548) animals; and in opossums in late embryo (E13.5), young postnatal (P0-P6), late postnatal (P10-P21), young adolescent (P28-P60), adolescent (P90-P120), and adult (P150-P180) animals (data from Cardoso-Moreira et al. (2019)). All used datasets and underlying data are shown in Supplementary table 14. Data from individual animals are shown as small dots, circles indicate mean values and error bars represent standard error of the mean (SEM). CDS – coding sequence, CPM – counts per million.

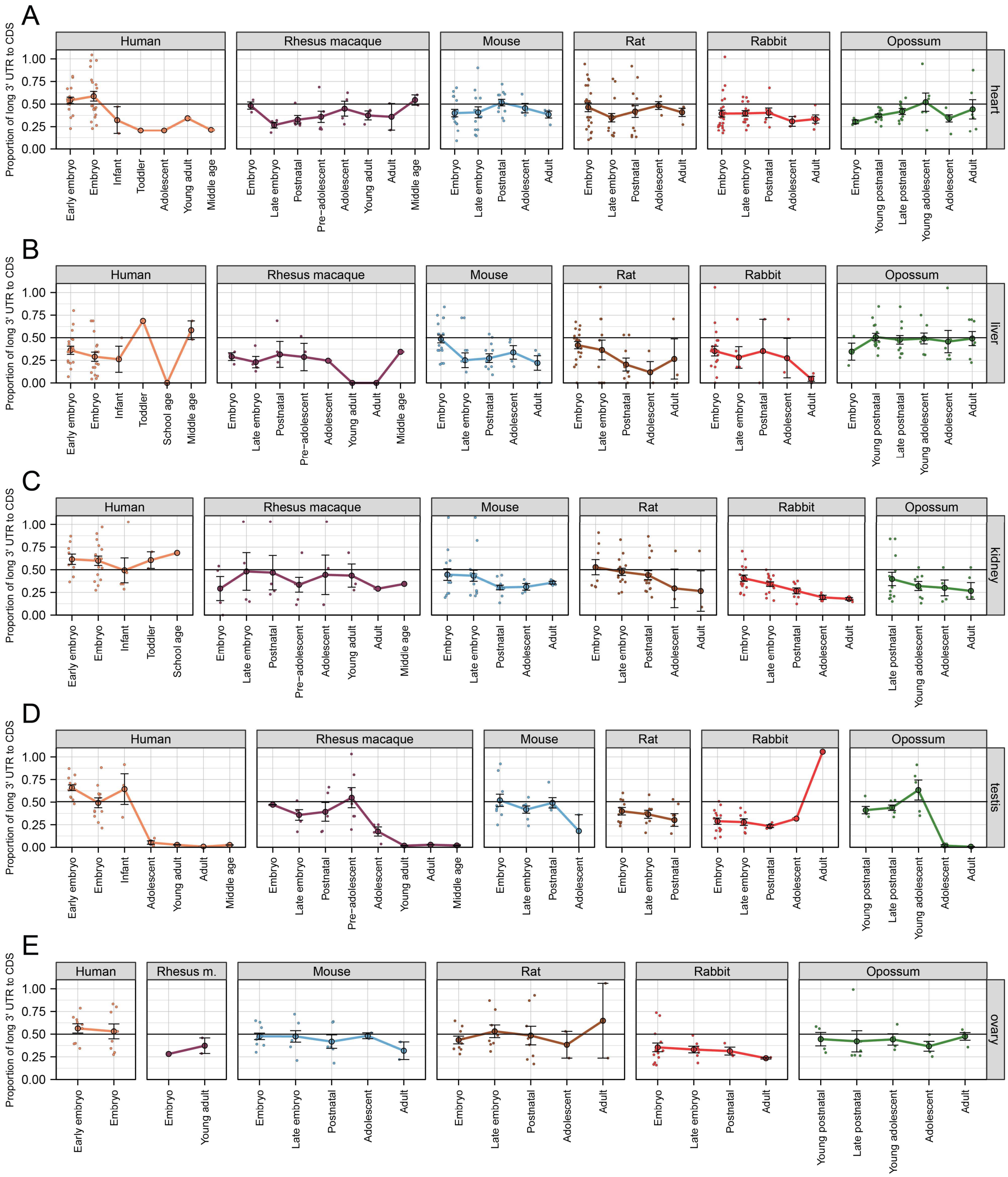

Supplementary figure S8. The proportion of *BDNF* transcripts with long 3' untranslated region in mammals' non-neural tissues. The ratio of *BDNF* transcripts with long 3' untranslated region (UTR) is shown in heart **(A)**, liver **(B)**, kidney **(C)**, testis **(D)**, and ovaries **(E)** relative to total *BDNF* levels measured by levels of *BDNF* coding sequence (CDS). All used datasets and underlying data are shown in Supplementary table 16. For more information on age classifications, see legend of Figure S7 (data from Cardoso-Moreira et al. (2019)). Data from individual animals are shown as small dots, circles indicate mean values and error bars represent standard error of the mean (SEM). CDS – coding sequence.

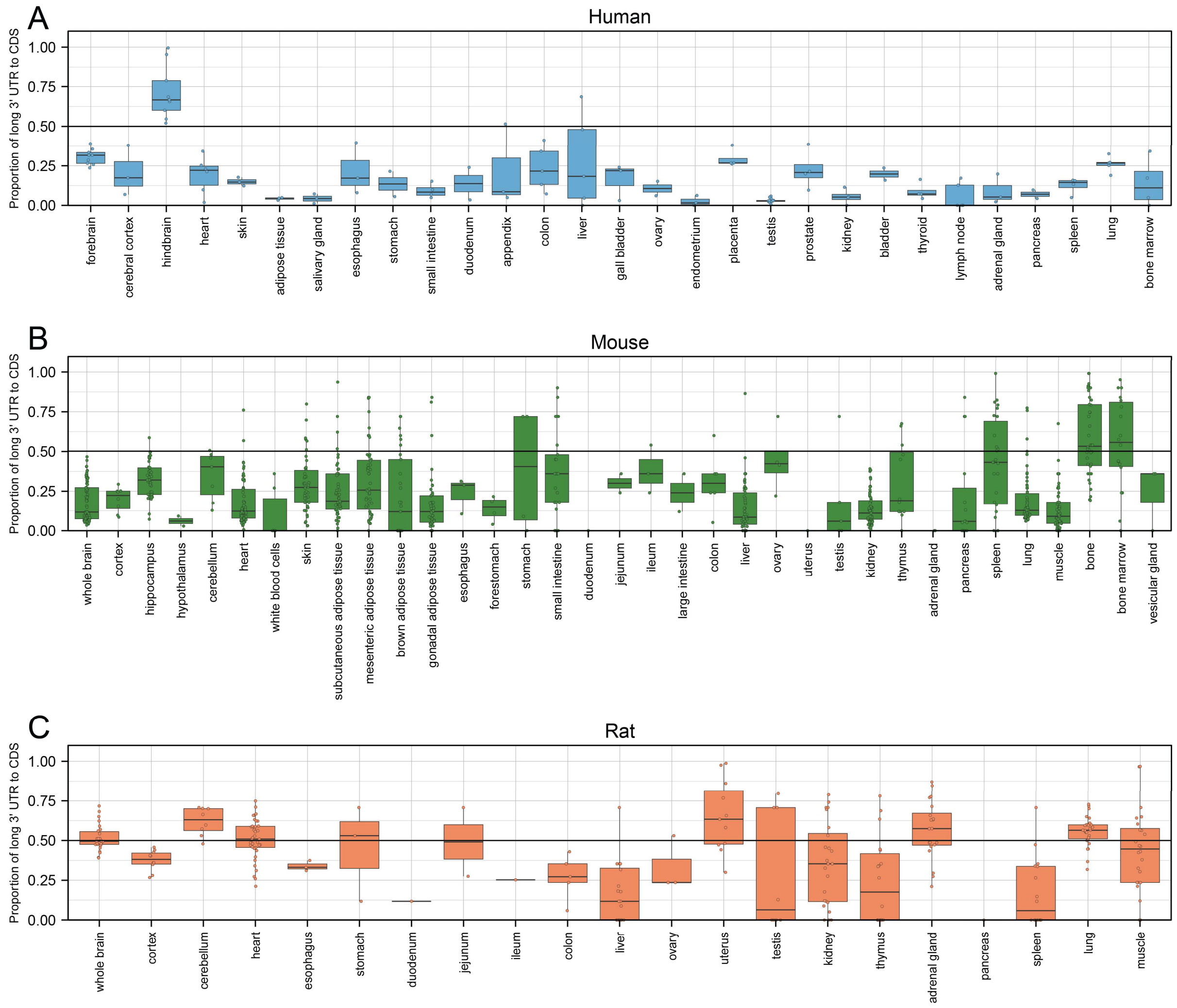

Supplementary figure S9. The ratios of *Bdnf* transcripts with long 3' untranslated region in adult human, mouse and rat brain regions and non-neural tissues. The ratio of *Bdnf* transcripts with long 3' untranslated region (UTR) is shown relative to total *Bdnf* levels measured by levels of *Bdnf* coding sequence (CDS) in adult humans **(A)**, or postnatal day 42+ mice **(B)** and rats **(C)**. All individual animals are shown as dots, the hinges show 25% and 75% quartiles, where the horizontal line shows the median value, the upper whisker extends from the hinge to the largest value no more than 1.5 of the inter-quartile range, and the lower whisker extends from the hinge to the smallest value at most 1.5 of the inter-quartile range of the hinge. All used datasets and underlying data are shown in Supplementary table 17. CDS – coding sequence.
